# Synthesis of organic liquid crystals containing selectively fluorinated cyclopropanes

**DOI:** 10.3762/bjoc.16.65

**Published:** 2020-04-14

**Authors:** Zeguo Fang, Nawaf Al-Maharik, Peer Kirsch, Matthias Bremer, Alexandra M Z Slawin, David O’Hagan

**Affiliations:** 1 School of Chemistry, Biomedical Sciences Research Complex, University of St Andrews, North Haugh, St Andrews, Fife KY16 9ST, United Kingdom; 2 Department of Chemistry, Faculty of Science, An Najah National University, Nablus, Palestine; 3 Merck KGaA, Liquid Crystal R&D Chemistry, Frankfurter Str. 250, 64293 Darmstadt, Germany

**Keywords:** dielectric anisotropy, difluorocarbene, organic liquid crystals, selectively fluorinated cyclopropanes

## Abstract

This paper describes the synthesis of a series of organic liquid crystals (LCs) containing selectively fluorinated cyclopropanes at their termini. The syntheses used difluorocarbene additions to olefin precursors, an approach which proved straightforward such that these liquid crystal candidates could be efficiently prepared. Their physical and thermodynamic properties were evaluated and depending on individual structures, they either displayed positive or negative dielectric anisotropy. The study gives some guidance into effective structure–property relationships for the design of LCs containing selectively fluorinated cyclopropane motifs.

## Introduction

**Figure 1:**
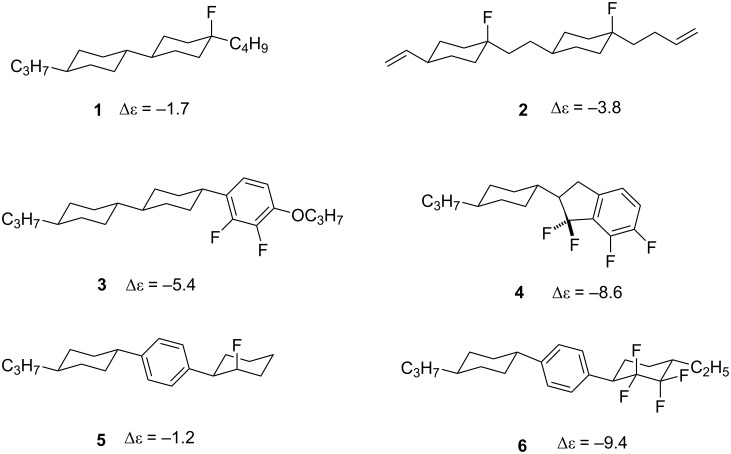
Examples of liquid crystal candidates with negative values for dielectric anisotropy (Δε) [6–10].

**Figure 2:**
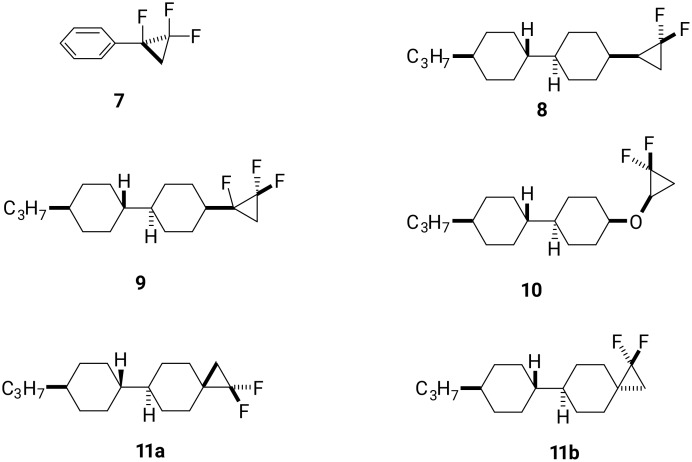
Synthetic candidate LC targets **8–11**.

Display materials associated with modern electronic devices such as personal mobile phones and TV sets, have changed dramatically in the past decades as liquid crystal display (LCD) technology has evolved [1–2]. A key challenge is to make suitable liquid crystalline (LC) materials that satisfy the requirements for different LCD technologies. For the traditional twisted nematic (TN) LCD technology, devices require liquid crystals with display positive dielectric anisotropy by which the molecular dipole moment is oriented parallel to the long axis of the molecule, while for the current vertical alignment (VA) LCD technology, liquid crystals with negative dielectric anisotropy are required [3]. Fluorine, as the most electronegative atom, forms stable bonds to carbon and can thus induce polarity. It is also attractive as a design feature due to the low polarizability of the C–F bond relative to other polar substituents such as nitrile and ester groups, and this reduces intermolecular interactions leading to lower viscosities and thus increased life time reliability [4–5]. A significant effort has been devoted to the development of liquid crystals with either positive or negative dielectric anisotropy by introducing the C–F bond or the dipole of a CF_2_ group either parallel or perpendicular to the long molecular axis. For example, in the area of negative liquid crystalline materials, Kirsch et al. have reported the synthesis of bicyclohexane and bis(cyclohexyl)ethane liquid crystals which contain axial fluorine substituents (Figure 1, **1** and **2**), however, due to the propensity for HF elimination from these molecules over time, these liquid crystals have not been adopted as commercial products [6–7]. 2,3-Difluoroaryl motifs such as **3** have proven to be a successful class of negative dielectric anisotropic liquid crystals and the more rigid 1,1,6,7-tetrafluoroindane **4** led to an enhanced polarity and improved negative dielectric anisotropy character [8]. Other liquid crystals of this class are the recently reported fluorinated cyclohexane **5** and 1,1,2,2-tetrafluorocyclohexane **6** [9–10], but the complexity of their synthesis has limited their development as practical materials.

As a consequence, it continues to be a research objective to introduce polar substituents in a manner that can influence the nature of the dielectric anisotropy of the resultant material. In this context we explore the cyclopropane motif containing fluorine atoms. Selectively fluorinated cyclopropanes have been widely used in pharmaceutical research [11], however, the introduction of fluorinated cyclopropanes into liquid crystal scaffolds has not received much attention. Haufe et al. [12], reported enantiomerically pure fluorinated diphenylcyclopropane carboxylates as potential chiral dopants to induce a cholesteric phase, but this was not extended to the exploration of positive or negative dielectrics. Recently, we reported the efficient synthesis of α,β,β-trifluorocyclopropanes **7** (Figure 2) through difluorocarbene addition to alpha-fluorostyrenes [13]. This used the extraordinary useful method for the generation of difluorocarbene from the Ruppert–Prakash (TMSCF_3_) reagent [14]. DFT analysis suggests the lowest energy conformer of aryl α,β,β-trifluorocyclopropane **7** orients the C–F bond perpendicular to the aryl ring. So, if the α,β,β-trifluorocyclopropane motif were introduced into an appropriate LC scaffold, it could reasonably induce negative dielectric anisotropy if the dipole associated with the cyclopropane remains oriented perpendicular to the molecular axis. Based on this idea, we report the design and synthesis of cyclopropanes **8** and **9** with an objective to assess their LC properties. Cyclopropane **8** with a single CF_2_ group offered a control to assess the influence of the alpha-fluorine atom in cyclopropane **9** containing three fluorines. In order to extend the study to explore other selectively fluorinated cyclopropane motifs we set out to prepare liquid crystal candidates **10** and isomers **11a** and **11b**. Ether **10** should have significant rotational freedom due to rotation around the ether bonds and will be compared with isomers **11a** and **11b** which are much more conformationally constrained (Figure 2).

Therefore, the aim of the study was to prepare these target liquid crystals and evaluate their properties relative to each other, to establish if there were any obvious structure–activity relationships to emerge for the design of positive or negative dielectric materials. Also the fixed spiro CF_2_-containing cyclopropanes **11a** and **11b** became targets to assess the impact of a configurationally rigid bicyclic motif. It is relatively obvious that for each of these isomers the CF_2_ dipole in each cyclopropane is orientated either along the molecular axis (for **11a**) or perpendicular to the molecular axis (for **11b**), and these were anticipated at the outset to give positive and negative dielectric anisotropy values, respectively.

## Results and Discussion

### Synthesis of liquid crystal candidates **8–11**

**Scheme 1:**
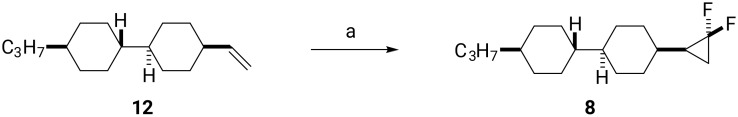
Synthesis of **8**. Reagents and conditions: a) TMSCF_3_, NaI, THF, reflux, 55% [13–14].

The synthesis of cyclopropane **8** started from olefin **12** (supplied by Merck KGaA, Darmstadt), as illustrated in Scheme 1. Difluorocyclopropane **8** can be directly prepared through a [2 + 1] carbene cycloaddition reaction. Thus, treatment of **12** with trimethyl(trifluoromethyl)silane (TMSCF_3_) and sodium iodide under refluxing conditions [13–14] gave the corresponding product **8** in one step and a 55% yield.

**Scheme 2:**
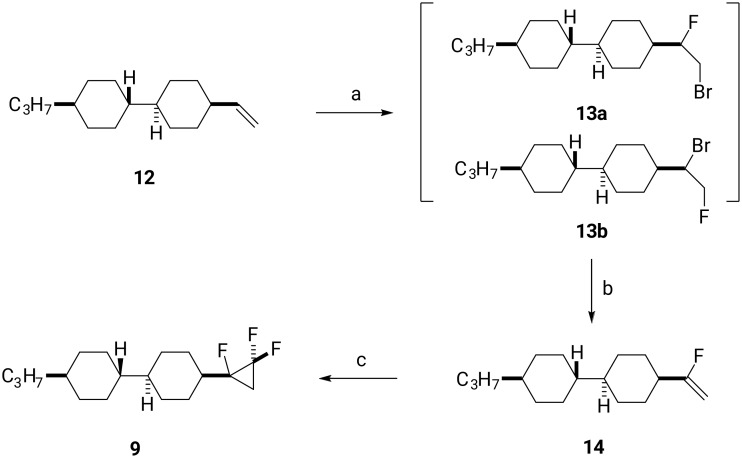
Synthesis of **9**. Reagents and conditions: a) NBS, HF·Py, DCM; b) *t*-BuOK, DCM, 42% in two steps [15]; c) TMSCF_3_, NaI, THF, reflux, 46%.

The synthesis of trifluorocyclopropane **9** is shown in Scheme 2. Compound **9** was prepared through bromofluorination of **12**, followed by base-induced HBr elimination [15], and then difluorocarbene addition to generate **9**. The starting olefin **12** was exposed to an excess of *N*-bromosuccinimide (NBS) and HF·Py to generate **13a** and **13b** as a mixture of regioisomers in a ratio of 4:1. There was no requirement to separate these isomers at this stage. Subsequent addition of potassium *tert*-butoxide into a mixture of **13a** and **13b** in dichloromethane (DCM) resulted in an efficient elimination to generate vinyl fluoride **14** as a major product. Vinyl fluoride **14** was purified by column chromatography in a 42% yield over the two steps. Finally, difluorocarbenene (:CF_2_) addition using the TMSCF_3_/NaI protocol was used to generate the target product **9** in 46% yield.

**Scheme 3:**
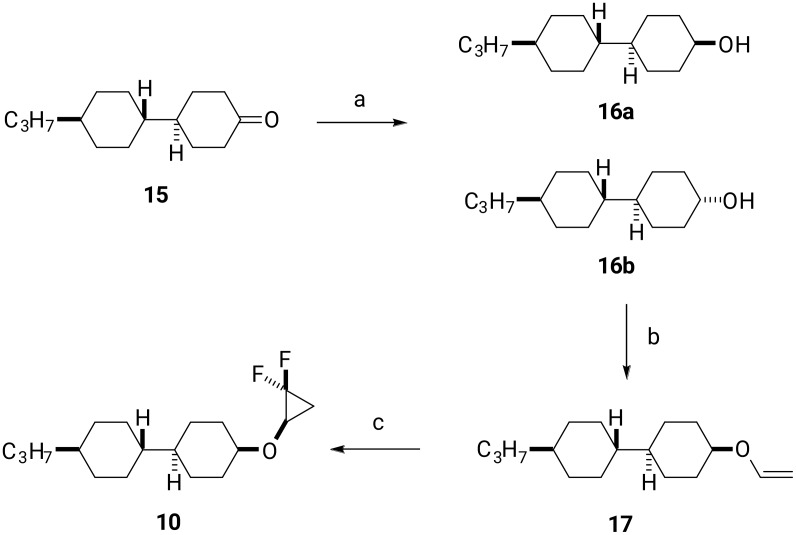
Synthesis of compound **10**. Reagents and conditions: a) NaBH_4_, MeOH, rt, 45%; b) C_4_H_9_OCH=CH_2_, Pd(TFA)_2_, BPhen, Et_3_N, 75 °C, 62% [16]; c) TMSCF_3_, NaI, THF, reflux, 50%.

The preparation of cyclopropyl ether **10** was accomplished as illustrated in Scheme 3. Reduction of cyclohexanone **15** with NaBH_4_ gave cyclohexanol **16** in a ratio of 2:1. The major *trans* product **16a** was purified as a single entity by column chromatography and was isolated in 45% yield. Vinyl ether **17** could be efficiently prepared using the methodology developed by Bosch [16]. Accordingly, treatment of **16a** with butyl vinyl ether, bathophenanthroline (BPhen) and Et_3_N, using palladium(II) trifluoroacetate as a catalyst, gave vinyl ether **17** in 62% yield in a single step. Finally, difluorocarbene-mediated difluorocyclopropanation generated **10** in 50% yield.

**Scheme 4:**
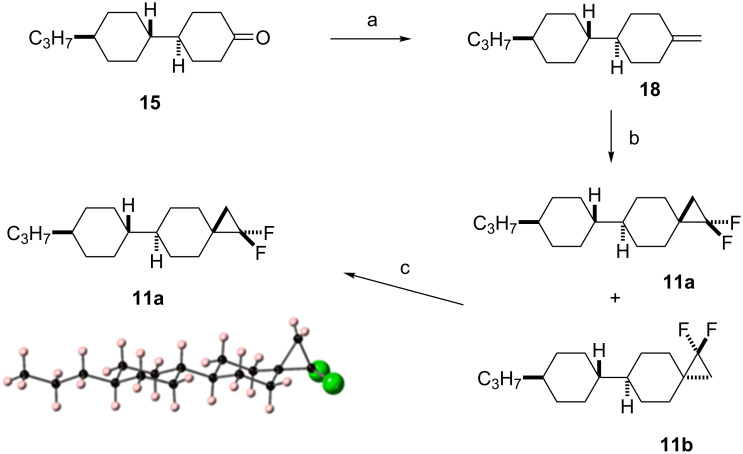
Synthesis of compounds **11**. Reagents and conditions: a) PPh_3_CH_3_Br, *t*-BuOK, diethyl ether, 0 °C to rt, 72% [17]; b) TMSCF_3_, NaI, THF, reflux; c) recrystallization, 27%.

The synthesis of the diastereoisomers of spiro LC candidates **11a** and **11b** also started from ketone **15** as illustrated in Scheme 4**.** Treatment of ketone **15** with methylenetriphenylphosphine which was generated in situ from methyltriphenylphosphonium bromide (PPh_3_CH_3_Br), generated *exo*-methylene cyclohexane **18** in a very straightforward manner, and the product could be isolated by chromatography in 72% yield [17]. This olefin was then subject to a difluorocarbene cyclopropanation using TMSCF_3_ and NaI, and this generated both isomers, **11a** and **11b**, in a ratio of 5:1, respectively. After three recrystallizations, the major isomer **11a** could be isolated as a single stereoisomer. It proved impossible to isolate a sample of isomer **11b** without a significant and dominant contamination with **11a**. Thus, it was with some frustration that we were unable to prepare a suitable sample, both in quantity and purity, of isomer **11b** for subsequent analysis. Stereoisomer **11a** was a crystalline solid and a suitable crystal was subject to X-ray structure analysis and the structure is shown in Scheme 4. In this way the stereochemistry of **11a** and **11b** could be confirmed unambiguously.

### Evaluation of liquid crystal candidates **8–11a**

With the four difluorocyclopropanes **8**–**11a** in hand, our attention focused on an experimental evaluation of their thermodynamic and physical properties, such as birefringence (Δ*n*) and dielectric anisotropy (Δε). DSC and POM analysis (Table 1) indicated that only compounds **8** and **9** exhibited smectic phases. The smectic phase of LC **8** was maintained over a broad temperature range (54–84 °C), whereas that for **10** and **11a** only showed a melting point and these compounds did not display any obvious liquid crystallinity (Table 1). Therefore, any development of these latter materials would require extended core structures to enhance their LC potential.

**Table 1:** Phase transition temperatures of compounds **8**–**11**.

	Phase transfer temperature [°C]

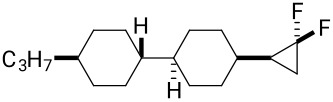 **8**	C 54 S_m_B 84 I
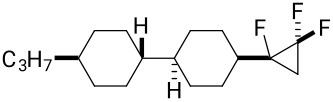 **9**	Tg −70 S_m_B 82 I^a^
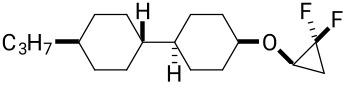 **10**	C35 I^a^
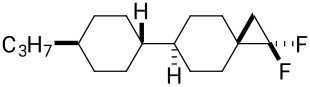 **11a**	C 42 I

^a^Decomposition (C = crystalline, S_m_B = smectic B, I = isotropic, Tg = glass transition temperature).

The birefringence Δ*n* (Table 2) for compounds **8**–**11a** was measured (Abbe refractometer). In all cases the Δ*n* values were low, which is entirely consistent with their core aliphatic structures, unlike materials with aromatic rings. The differences for Δ*n* for **8**, **9** and **10**, were found to be small, whereas the spiro-cyclopropane **11a** had a significantly, decreased Δ*n*.

**Table 2:** Physical properties of compounds **8**–**11**.

Compound	Δ*n*	Δε

**8**^a^	0.056	−0.5
**9**^b^	0.055	−0.2
**10**^b^	0.063	1.3
**11a**^a^ **11a**^c^ **11b^c^**	0.036 0.054 0.051	4.8 4.3 −1.1

^a^Δ*n* were extrapolated from a 10 wt % solution in liquid crystal mixture Merck ZLI-4792. Δε were extrapolated from a 10 wt % solution in liquid crystal mixture Merck Merck ZLI-2857. ^b^Δ*n* were extrapolated from a 5 wt % solution in liquid crystal mixture Merck ZLI-4792. Δε were extrapolated from a 10 wt % solution in liquid crystal mixture Merck Merck ZLI-2857. ^c^Calculated data only at AM1//B3LYP/6-31G(d) level [18].

As discussed, either positive or negative dielectric anisotropy is an important parameter related to the driving voltage of LC displays and in order to reduce energy consumption, good materials should induce a large value for dielectric anisotropy. Compounds **8** and **9** were found to exhibit the expected negative Δε values, however, the overall polarity is low (**8** = −0.5, **9** = −0.2). This is presumably due to the ability of the C–C bond attached to cyclopropane to rotate, and thus the compound will adopt conformations with low overall molecular polarities. A comparison of the Δε for **8** and **9** illustrates that incorporation of the third alpha*-*fluorine lowers the overall Δε value. This is presumably due to a resulting decrease in the overall molecular dipole moment (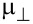) along the minor axis. When an ether was used as the linkage in compound **10**, the compound becomes more flexible and, perhaps surprisingly, the overall Δε parameter became more positive. Clearly the effect of introducing a C–O bond renders this molecule dissimilar from the others in this series. However, changing the core aliphatic structure to the more rigid spiro structure **11a**, brought about a dramatic enhancement of the molecular dipole moment (μ_||_), resulting in a much more positive and increased parameter value (Δε = 4.8). This experimental value was close to the predicted calculated value (Δε = 4.3) as shown in Table 2. It is clear that there is an advantage in a rigid structure for imparting a maximum polarity. As stated above, we were unable to obtain a stereochemically pure sample of diastereoisomer **11b**, free from **11a**, however, theory indicates that **11b** should display negative dielectric anisotropy, although the magnitude of the parameter was relatively low (Δε = −1.1). Thus the two isomers constitute two different classes of LC materials, entirely as a consequence of stereochemistry.

**Figure 3:**
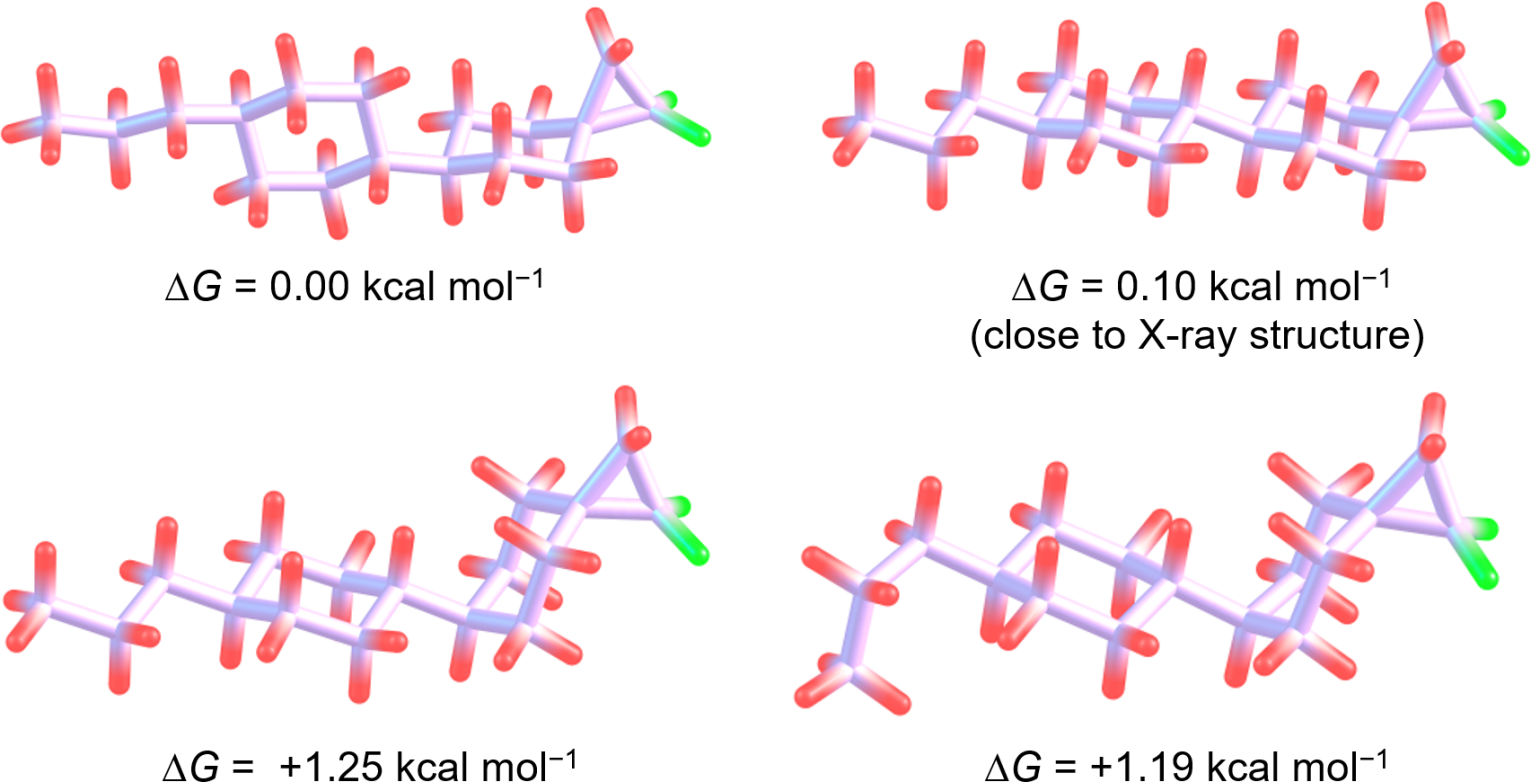
Theory study exploring the relative energies for different conformers of **11a**.

The best compound of this series is **11a** and it was of interest to get some insight into its conformational flexibility. Thus, a conformational analysis of **11a** (and **11b**) was conducted computationally (ωB97X-D/6-311+G(2d,p)//ωB97X-D/6-31G(d) + ZPE level (see Supporting Information File 1)). There are many potential low energy conformations arising from alkyl side chain C–C bond rotations which would not be anticipated to change the overall properties significantly, however, we were interested in assessing the energy required for the bispirocyclohexane ring to adopt an axial rather than the lower energy equatorial arrangement. For **11a**, the lowest energy conformer found was a rotamer around the central C–C bond linking the cyclohexane rings, but an essentially isoenergetic conformer is that found in the X-ray crystal structure. The first conformers where the terminal bispirocyclohexane ring lies axial are significantly higher in energy (Δ*G* = +1.25 kcal/mol^−1^ and Δ*G* = +1.19 kcal/mol^−1^) than the equatorial conformers as illustrated in Figure 3. Notably for both the axial and equatorial conformers, the orientation of the CF_2_ group, and therefore its dipole, remains perpendicular to the long molecular axis (requirement for +ve dielectric). A similar result was obtained for stereoisomer **11b** (see Supporting Information File 1) again with the first axial conformer at Δ*G* = +1.14 kcal/mol^−1^ above the ground state equatorial conformer. In this case the orientation of the CF_2_ group, and therefore the dipole, remains perpendicular to the long molecular axis (requirement for −ve dielectric) for both the axial and equatorial conformers. This analysis, and that of the X-ray structure, suggests that the equatorial conformers will dominate in condensed phases, and that the overall molecular dipoles will not be compromised by minor contributions of axial conformers.

## Conclusion

In conclusion, a series of candidate liquid crystals **8–11a** with difluorocyclopropane motifs at their termini were prepared in a straightforward manner. In all cases the cyclopropane was introduced by difluorocarbene addition to an olefin, the difluorocarbene being generated from TMSCF_3_/NaI. An evaluation of the physical properties of LC candidates **8**–**11a** revealed that LC’s **8** and **9** exhibited smectic phases with low levels of negative dielectric anisotropy. The relatively high positive dielectric anisotropy of compound **11a** indicates that the configurational constrained motif is required to increase the magnitude of the Δε parameter. Although it proved challenging to isolate a stereochemically clean sample of isomer **11b** for experimental evaluation, theory predicts that it will have a modest negative dielectric value, indicating the importance of stereochemistry in dictating the class of LC material. We conclude too that the overall performance of prototype LCs **11a** and **11b** should not be compromised by conformational flexibility. Although none of the compounds prepared here met all of the criteria for commercial development, the study gives an insight into the potential for this novel difluorocyclopropane motif in liquid crystal design and development.

## Supporting Information

File 1 Experimental protocols and NMR spectra.

File 2 Crystallographic information file of compound **11a**.

## References

[1] Kirsch P (2013). Modern Fluoroorganic Chemistry.

[2] Kirsch P, Bremer M (2000). Angew Chem, Int Ed.

[3] Hird M (2007). Chem Soc Rev.

[4] O'Hagan D (2008). Chem Soc Rev.

[5] Bremer M, Kirsch P, Klasen-Memmer M, Tarumi K (2013). Angew Chem, Int Ed.

[6] Kirsch P, Tarumi K (1998). Angew Chem, Int Ed.

[7] Kirsch P, Heckmeier M, Tarumi K (1999). Liq Cryst.

[8] Bremer M, Lietzau L (2005). New J Chem.

[9] Al-Maharik N, Kirsch P, Slawin A M Z, Cordes D B, O'Hagan D (2016). Org Biomol Chem.

[10] Yamada S, Hashishita S, Konishi H, Nishi Y, Kubota T, Asai T, Ishihara T, Konno T (2017). J Fluorine Chem.

[11] Fang Z, Cordes D B, Slawin A M Z, O'Hagan D (2019). Chem Commun.

[12] Hruschka S, Fröhlich R, Kirsch P, Haufe G (2007). Eur J Org Chem.

[13] Thomson C J, Zhang Q, Al-Maharik N, Bühl M, Cordes D B, Slawin A M Z, O’Hagan D (2018). Chem Commun.

[14] Wang F, Luo T, Hu J, Wang Y, Krishnan H S, Jog P V, Ganesh S K, Prakash G K S, Olah G A (2011). Angew Chem, Int Ed.

[15] Meyer O G J, Fröhlich R, Haufe G (2000). Synthesis.

[16] Bosch M, Schlaf M (2003). J Org Chem.

[17] Conner M L, Brown M K (2016). J Org Chem.

[18] Dewar M J S, Zoebisch E G, Healy E F, Stewart J J P (1985). J Am Chem Soc.

