# Supplementary material for: Synthesis of organic liquid crystals containing selectively fluorinated cyclopropanes
Source: Beilstein J Org Chem. 2020 Apr 14;16:674–80. doi: 10.3762/bjoc.16.65 (PMC7176930; doi:10.3762/bjoc.16.65)

BEILSTEIN JOURNAL OF ORGANIC CHEMISTRY

Supporting Information

for

Synthesis of organic liquid crystals containing selectively fluorinated cyclopropanes

Zeguo Fang, Nawaf Al-Maharik, Peer Kirsch, Matthias Bremer, Alexandra M. Z. Slawin and David O'Hagan

Beilstein J. Org. Chem. **2020,** *16,* 674–680. doi:10.3762/bjoc.16.65

Experimental protocols and NMR spectra

License and Terms: This is a supporting information file under the terms of the Creative Commons Attribution License (http://creativecommons.org/licenses/by/4.0). Please note that the reuse, redistribution and reproduction in particular requires that the authors and source are credited.
The license is subject to the *Beilstein Journal of Organic Chemistry* terms and conditions: (https://www.beilstein-journals.org/bjoc)

Contents

General information...S1

Synthetic procedure, analytical and NMR data...S2

NMR spectra...S10

Computational study...S22

Single crystal X-ray structure analysis...S30

Images for DSC analysis and POM...S32

General information

All chemicals were purchased from Sigma-Aldrich or Fluorochem except the starting material **12** and **15**, which were supplied by Merck & Co. All the reactions were carried out in normal glassware under the atmosphere of argon, unless stated otherwise. The phase transition temperatures are given in °C. C = crystalline, Sx = smectic X, N = nematic, I = isotropic. For the pure substances the mesophases were identified by polarized optical microscopy, and the phase transition temperatures by differential scanning calorimetry (DSC). Δn was determined by linear extrapolation from Merck mixture ZLI-4792 (T_{NI} = 92.8 °C, Δε = 5.3, Δn = 0.0964). The dielectric anisotropy was extrapolated from the commercially available Merck mixture ZLI-2857 (T_{NI} = 82.3 °C, Δε = −1.4, Δn = 0.0776). The extrapolated values are corrected empirically for differences in the order parameter.

The progress of reactions was followed by thin-layer chromatography (TLC) using aluminium plates coated with silica gel ($60F_{245}$ Merck). TLC plates were examined under UV light at 254

and 266 nm. Column chromatography was performed on Merck Geduran silica gel (250–400 mesh) under a positive pressure of compressed air eluting with solvents as supplied.

Proton (^{1}H) and proton-decoupled nuclear magnetic resonance spectra ($^{19}F\{^{1}H\}$, $^{13}C\{^{1}H\}$) were recorded on a Bruker AV 300, Bruker AV 400, Bruker AVII 400, Bruker AVIII-HD 500 or Bruker AVIII 500 instrument. Chemical shifts are reported in parts per million (ppm). Tetramethylsilane (δ = 0 ppm) functioned as an internal standard for ^{1}H and ^{13}C NMR experiments. $CFCl_3$ was used as an external standard for ^{19}F NMR experiments. Coupling constants (*J*) are reported in Hz.

High-resolution mass spectrometry was acquired using electrospray ionisation (ESI), on a ThermoFisher Excalibur Orbitrap Spectrometer, operating in positive and negative mode, from solutions of the analyate in methanol or acetonitrile. Mass analyses were done at the University of St Andrews. Additional data was obtained at the EPSRC UK National Mass Spectrometry Facility at Swansea University, UK.

X-ray crystal structures were obtained on a Rigaku XtaLAB P200 diffractometer, using multi-layer mirror monochromated Cu Kα radiation, at the University of St Andrews by Prof. Alexandra Slawin and Dr David Cordes. Data was analysed by using CrystalMaker.

Synthetic procedure

***trans*-4-(*trans*-4-Propylcyclohexyl)cyclohexanol 16a.** ^{1}**H NMR** (400 MHz, $CDCl_3$) δ_H 0.79-1.38 (m, 18 H), 1.61-1.84 (m, 7 H), 1.91-2.08 (m, 2 H), 3.51 (m, 1 H). Data is in agreement with the literature data.[1]

1. K. Kanie, S. Takehara, T. Hiyama. *Bull. Chem. Soc. Jpn.,* **2000**, *73*, 1875-1892.

4-(2,2-Difluorocyclopropyl)-4'-propyl-1,1'-bi(cyclohexane)

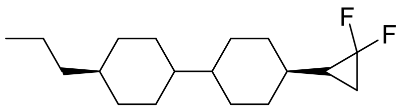

8

In a similar manner as already described[2]. To a solution 1'-propyl-4-vinyl-1,4'-bi(cyclohexane) (**12**, 100 mg, 0.43 mmol, 1 equiv) and $TMSCF_3$ (151.7 mg, 1.07 mmol, 2.5 equiv) in anhydrous THF, NaI (159 mg, 1.07 mmol, 2.5 equiv) was added. The resulting suspension was stirred at 75 °C under N_2 atmosphere. The reaction was monitored by NMR. After completion, the reaction mixture was allowed to cool to rt and solvent was removed in vacuo. The crude residue was diluted with diethyl ether (50 mL) and washed with distilled water (50 mL). The phases were separated and the aqueous layer was extracted with diethyl ether (2 × 50 mL). The combined organic phases were washed sequentially with saturated aqueous solutions of Na_2SO_3 and $NaHCO_3$, followed by drying over Na_2SO_4, filtration and evaporation of solvent in vacuo. Purification by flash column chromatography (petroleum ether) gave the desired product as the white solid (66.7 mg, 55%); ^{1}**H NMR** (400 MHz, $CDCl_3$) δ_H 0.62-1.42 (m, 22H), 1.60-2.02 (m, 8H). ^{19}**F {^{1}H} NMR** (376 MHz, $CDCl_3$) δ_F -126.6 (d, J = 155.6 Hz, 1F, CF_2), -145.0 (d, J = 155.6 Hz, 1F, CF_2); ^{19}**F NMR** (376 MHz, $CDCl_3$) δ_F -126.6 (m, 1F, CF_2), -145.0 (m, 1F, CF_2); ^{13}**C NMR** (125 MHz, $CDCl_3$) δ_C 114 (t, J = 284.0 Hz), 43.4, 42.9, 39.8, 37.6, 37.0, 33.6, 33.4, 32.1, 30.1, 29.7, 29.5, 28.7 (t, J = 10.4 Hz), 20.0, 15.2 (t, J = 10.7 Hz),14.5; **HRMS** (ASAP$^+$) m/z calcd for $C_{18}H_{30}F_2$ 284.2316, found 284.2313.

2. Thomson, C. J.; Zhang, Q.; Al-Maharik, N.; Bühl, M.; Cordes, D. B.; Slawin, A. M. Z.; O'Hagan, D. *Chem. Commun.* **2018,** *54*, 8415–8418.

4-(1-Fluorovinyl)-4'-propyl-1,1'-bi(cyclohexane)

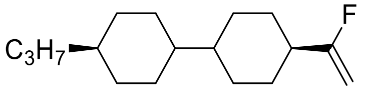

14

To a solution 1'-propyl-4-vinyl-1,4'-bi(cyclohexane) (**12**, 2.1 g, 8.97 mmol, 1 equiv) in anhydrous DCM, NBS (3.24 g, 17.9 mmol, 2 equiv) and HF·Py (0.48 g, 17.9 mmol, 2 equiv) were added under N_2 atmosphere at 0 °C. The reaction mixture was warmed to rt, and then stirred overnight. After completion, the resulting solution was extracted with CH_2Cl_2, the combined organic phases were washed by aqueous solution of $NaHCO_3$, then dried under vacuo. *t*-BuOK (4.03 g, 35.88 mmol, 4 equiv) was added to the mixture in CH_2Cl_2 and the colour of mixture became brown. The reaction mixture was monitored by NMR. After the completion, the reaction mixture was slowly poured into dilute HCl solution at 0 °C and stirred for 10 minutes. The resulting solution was extracted with CH_2Cl_2 (3 × 50 mL) and the combined organic phases were washed sequentially with aqueous dilute HCl (0.1 M, 50 mL) and a saturated aqueous solution of $NaHCO_3$ (50 mL), followed by drying over Na_2SO_4. After filtration, solvent was removed in vacuo. Purification by flash column chromatography (petroleum ether) afforded the product as the oil (0.949 g, 42%). **^{1}H NMR** (400 MHz, $CDCl_3$) δ_H 4.42 (dd, *J* = 18.6, 2.8 Hz, $CFCH_2$, 1H), 4.16 (ddd, *J* = 51.6, 2.8, 0.9 Hz, $CFCH_2$, 1H), 2.08-1.89 (m, 3H), 1.81-1.66 (m, 6H), 1.36-1.09 (m, 7H), 1.07-0.90 (m, 6H), 0.90-0.79 (m, 5H); **^{19}F {^{1}H} NMR** (376 MHz, $CDCl_3$) δ_F -99.0 (s, 1F, CHF); **^{19}F NMR** (376 MHz, $CDCl_3$) δ_F -99.0 (ddd, *J* = 51.6, 18.6, 12.5 Hz, 1F, CHF); **^{13}C NMR** (125 MHz, $CDCl_3$) δ_C 171.1 (d, *J* = 257.9 Hz), 87.1 (d, *J* = 20.8 Hz), 43.3, 42.8, 40.6 (d, *J* = 25.1 Hz), 39.8, 37.6, 33.5, 30.1 (d, *J* = 2.5 Hz), 30.0, 29.4, 20.0, 14.5; HRMS (EI$^+$) m/z calcd for $C_{17}H_{29}F$ 252.2247, found 252.2235.

4-Propyl-4'-(1,2,2-trifluorocyclopropyl)-1,1'-bi(cyclohexane)

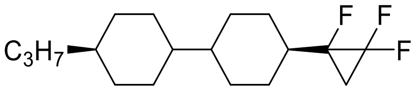

9

In a similar manner as already described[2]. To a solution of **14** (0.9 g, 3.57 mmol, 1 equiv) and $TMSCF_3$ (1.26 g, 8.93 mmol, 2.5 equiv) in anhydrous THF, NaI (1.33 g, 8.93 mmol, 2.5 equiv) was added. The resulting suspension was stirred at 75 °C for 20 h under N_2 atmosphere. After completion, the reaction mixture was allowed to cool to rt and solvent was removed in vacuo. The crude residue was diluted with diethyl ether (50 mL) and washed with distilled water (50 mL). The phases were separated and the aqueous layer was extracted with diethyl ether (2 × 50 mL). The combined organic phases were washed sequentially with saturated aqueous solutions of Na_2SO_3 and $NaHCO_3$, followed by drying over Na_2SO_4, filtration and evaporation of solvent in vacuo. Purification by flash column chromatography (petroleum ether) gave the desired product as the white solid (496 mg, 46%) **^{1}H NMR** (400 MHz, $CDCl_3$) δ_H 1.85-1.67 (m, 9H), 1.47-1.26 (m, 6H), 1.17-0.93 (m, 9H), 0.88-0.80 (m, 5H); **^{19}F {^{1}H} NMR** (376 MHz, $CDCl_3$) δ_F -140.2 (dd, *J* = 169.5, 13.6 Hz, 1F, CF_2), -140.8 (d, *J* = 169.5, Hz, 1F, CF_2), -202.6 (d, *J* = 13.6 Hz, CHF); **^{19}F NMR** (376 MHz, $CDCl_3$) δ_F -140.2 (m, 1F, CF_2), -140.8 (ddd, *J* = 169.5 Hz, 17.6, 6.7 Hz, 1F, CF_2), -202.6 (m, CHF); **^{13}C NMR** δ_C 110.6 (dt, *J* = 294.8, 10.0 Hz, CF_2), 81.2 (ddd, *J* = 238.5, 12.7, 8.8 Hz, CHF), 43.2, 42.6, 39.8, 38.7 (d, *J* = 19.2 Hz), 37.6, 33.6, 29.6, 29.5, 29.4, 28.1, 27.0, 21.2 (m), 20.0, 14.4; HRMS (ASAP$^+$) m/z calcd for $C_{18}H_{29}F_3$ 302.2221, found 302.2217.

4-Propyl-4'-(vinyloxy)-1,1'-bi(cyclohexane)

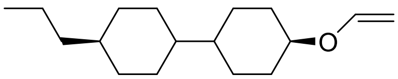

17

Palladium trifluoroacetate (96 mg, 0.29 mmol, 0.05 equiv) and 4,7-diphenylphenanthroline (96 mg, 0.29 mmol, 0.05 equiv) were dissolved in butyl vinyl ether (11.6 g, 116 mmol, 20 equiv). Alcohol **16a** (1,3 g, 5.8 mmol, 1 equiv) and triethylamine (0.058 mg, 0.029 mmol, 0.1 equiv) were added to the yellow solution, the flask sealed and stirred at 75 °C for 13 h. After completion, the resulting solution was extracted with CH_2Cl_2 (3 × 50 mL) and the combined organic phases were washed sequentially with water (2 × 50 mL), followed by drying over Na_2SO_4. After filtration, solvent was removed in vacuo. Purification by flash column chromatography (petroleum ether) afforded the product as the colourless liquid (900 mg, 62%). **^{1}H NMR** (400 MHz, $CDCl_3$) δ_H 6.33 (dd, *J* = 14.1, 6.6 Hz, 1H), 4.28 (dd, *J* = 14.1, 1.4 Hz, 1H), 3.97 (dd, *J* = 6.6, 1.4 Hz, 1H), 3.67-3.57 (m, 1H), 2.11-2.01 (m, 2H), 1.80 - 1.69 (m, 6H), 1.35-1.21 (m, 5H), 1.18 – 1.08 (m, 3H), 1.08 – 0.76 (m, 10H); **^{13}C NMR** (125 MHz, $CDCl_3$) δ_C 150.7, 88.0, 78.8, 42.8, 42.3, 39.8, 37.5, 33.5, 32.3, 30.2, 27.8, 20.0, 14.4; **HRMS** (ES) m/z calcd for $[M+H]^+$ $C_{17}H_{31}O$ 251.2366, found 251.2369.

4-(2,2-Difluorocyclopropoxy)-4'-propyl-1,1'-bi(cyclohexane)

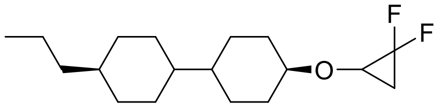

10

In a similar manner as already described[2]. To a solution alkene **17** (490 mg, 1.96 mmol, 1 equiv) and $TMSCF_3$ (556.6 mg, 3.92 mmol, 2.0 equiv) in anhydrous THF, NaI (584 mg, 3.92 mmol, 2.0 equiv) was added. The resulting suspension was stirred at 75 °C for 20 h under N_2 atmosphere. After completion, the reaction mixture was allowed to cool to rt and solvent was removed in vacuo. The crude residue was diluted with diethyl ether (50 mL) and washed with distilled water (50 mL). The phases were separated and the aqueous layer was extracted with diethyl ether (2 × 50 mL). The combined organic phases were washed sequentially with saturated aqueous solutions of Na_2SO_3 and $NaHCO_3$, followed by drying over Na_2SO_4, filtration and evaporation of solvent in vacuo. Purification by flash column chromatography (petroleum ether) gave the desired product as the white solid (294 mg, 50%). **^{1}H NMR** (400 MHz, $CDCl_3$) δ_H 3.63 (m, 1H), 3.37 (m, 1H), 2.08 (m, 2H), 1.82-1.65 (m, 6H), 1.54-0.77 (m, 20H); **^{19}F {^{1}H} NMR** (376 MHz, $CDCl_3$) δ_F -130.7 (d, *J* = 164.4 Hz, 1F, CF_2), -147.4 (d, *J* = 164.4 Hz, 1F, CF_2); **^{19}F NMR** (376 MHz, $CDCl_3$) δ_F -130.7 (m,1F, CF_2), -147.4 (m, 1F, CF_2); **^{13}C NMR** (125 MHz, $CDCl_3$) δc 111.5 (t, *J* = 289.5 Hz), 79.9, 54.9 (dd, *J* = 13.6, 9.3 Hz), 42.8, 42.4, 39.7, 37.5, 33.5, 32.3, 32.0, 30.1, 28.0, 27.9, 20.0, 18.1 (t, *J* = 10.3 Hz), 14.4; **HRMS** (ASAP) m/z calcd [M-H] $C_{18}H_{29}F_2O$ 299.2187, found 299.2183, calcd. [M] 300.2220, found 300.2230.

4-Methylene-4'-propyl-1,1'-bi(cyclohexane)

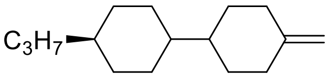

18

To a flame-dried flask were added ketone **15** (1.0 g, 4.5 mmol, 1.0 equiv), methyltriphenylphosphonium bromide (1.74 g, 5.4 mmol, 1.2 equiv) and diethyl ether (50 mL). Potassium *tert*-butylate (0.6 g, 5.4 mmol, 1.2 equiv) was added down the walls of the flask. The reaction mixture was allowed to stir at room temperature for 6 h. After completion, the crude residue was diluted with diethyl ether (50 mL) and washed with distilled water (50 mL). The phases were separated and the aqueous layer was extracted with diethyl ether (2 × 50 mL). The combined organic phases were washed sequentially with saturated aqueous solutions of Na_2SO_3 and $NaHCO_3$, followed by drying over Na_2SO_4, filtration and evaporation of solvent in vacuo. Purification by flash column chromatography (petroleum ether) gave the desired product as the liquid (714 mg, 72%). **^{1}H NMR** (400 MHz, $CDCl_3$) δ_H 4.57 (t, *J* = 1.7 Hz, 2H), 2.45-2.19 (m, 2H), 2.08-1.88(m, 2H), 1.83-1.61 (m, 7H), 1.37-0.76 (m, 15H); **^{13}C NMR** (101 MHz, $CDCl_3$) δc 150.5, 106.2, 42.94, 42.91, 39.8, 37.6, 35.0, 33.6, 31.5, 30.1, 20.0, 14.4; **HRMS** (EI^+) m/z calcd [M] $C_{16}H_{28}$ 220.2191, found 220.2193.

1,1-Difluoro-6-(4-propylcyclohexyl)spiro[5.2]octane

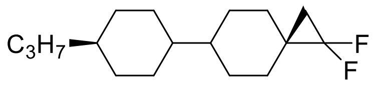

11a

In a similar manner as already described[2]. To a solution alkene **18** (730 mg, 3.31 mmol, 1 equiv) and $TMSCF_3$ (1.18 g, 8.29 mmol, 2.5 equiv) in anhydrous THF, NaI (1.24 g, 8.29 mmol, 2.5 equiv) was added. The resulting suspension was stirred at 75 °C for 20 h under N_2 atmosphere. After completion, the reaction mixture was allowed to cool to rt and solvent was removed in vacuo. The crude residue was diluted with diethyl ether (50 mL) and washed with distilled water (50 mL). The phases were separated and the aqueous layer was extracted with diethyl ether (2 × 50 mL). The combined organic phases were washed sequentially with saturated aqueous solutions of Na_2SO_3 and $NaHCO_3$, followed by drying over Na_2SO_4, filtration and evaporation of solvent in vacuo. Purification by flash column chromatography (petroleum ether) afforded the stereoisomers. After recrystallisation (petroleum ether/ethyl acetate = 5:1) for three times, the desired product was obtained as the white solid (241 mg, 27%). **^{1}H NMR** (400 MHz, $CDCl_3$) δ_H 1.80-1.56 (m, 8H), 1.40-1.24 (m, 4H), 1.19-0.87 (m, 16H); **^{19}F {^{1}H} NMR** (376 MHz, $CDCl_3$) δ_F -139.1 (s, 2F, CF_2); **^{19}F NMR** (376 MHz, $CDCl_3$) δ_F -139.1 (t, *J* = 8.3 Hz, 2F, CF_2); **^{13}C NMR** δ_C 116.9 (t, J = 287.7 Hz), 43.0, 42.9, 39.8, 37.6, 33.5, 30.1, 29.5 (t, *J* = 10.7 Hz), 28.8, 27.5, 21.7 (t, J = 10.0 Hz), 20.1, 14.5; **HRMS** (EI^+) m/z calcd. [M] $C_{17}H_{28}F_2$ 270.2159, found 270.2157.

NMR spectra

^{1}H NMR 8

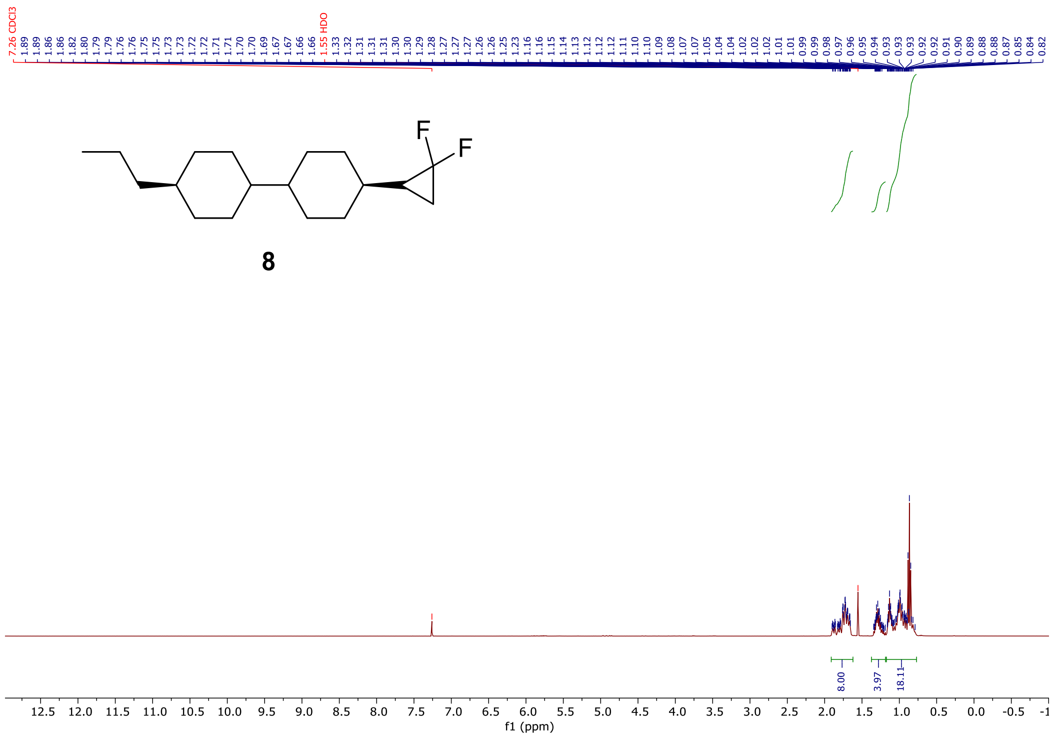

^{19}F {^{1}H} NMR 8

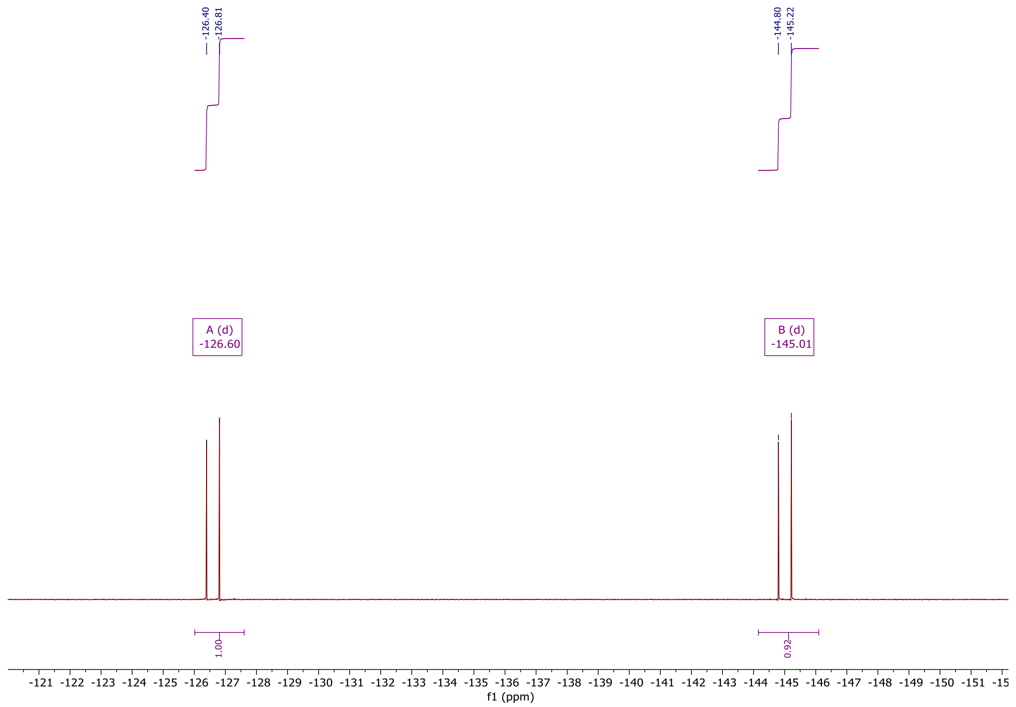

DEPT ^{13}C NMR 8

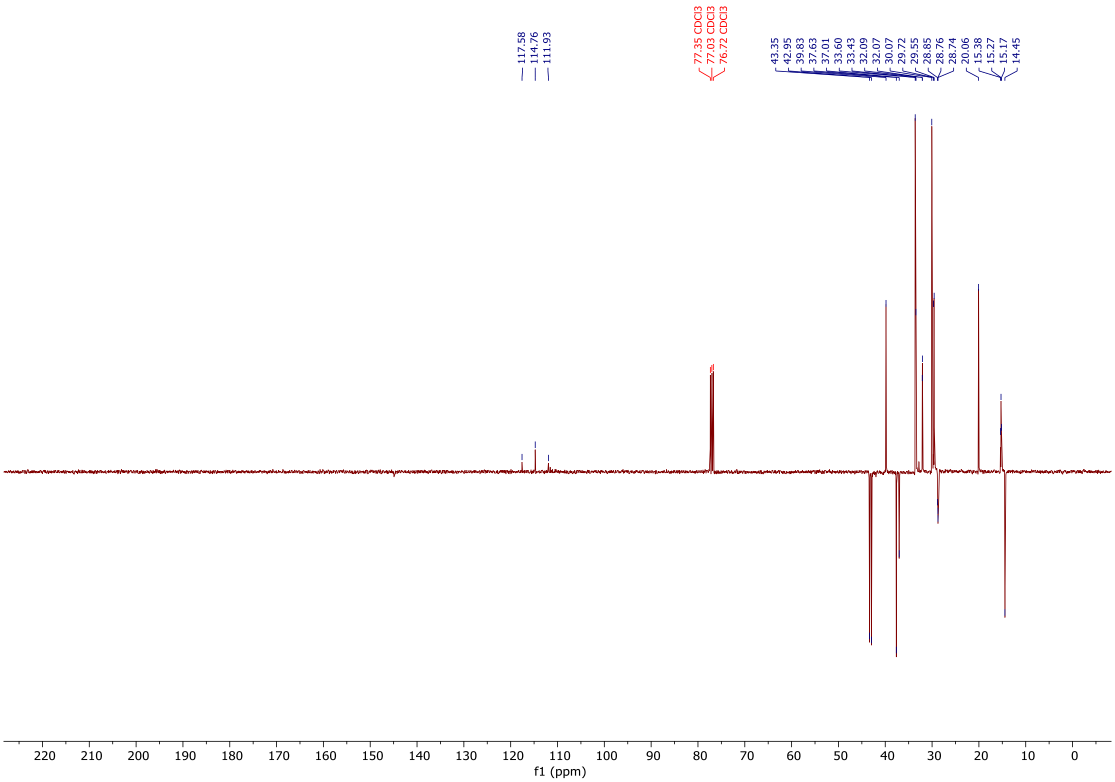

^{1}H NMR 14

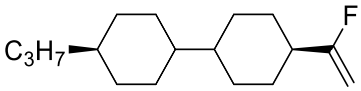

14

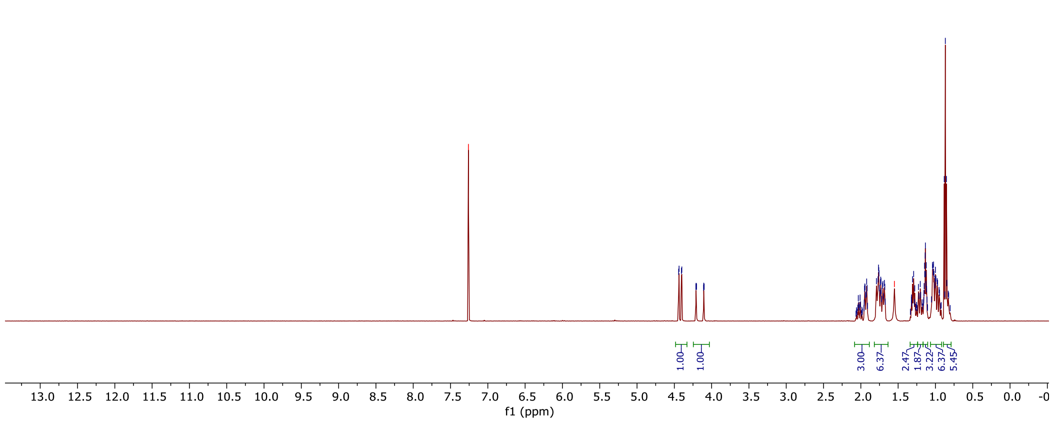

^{19}F {^{1}H} NMR 14

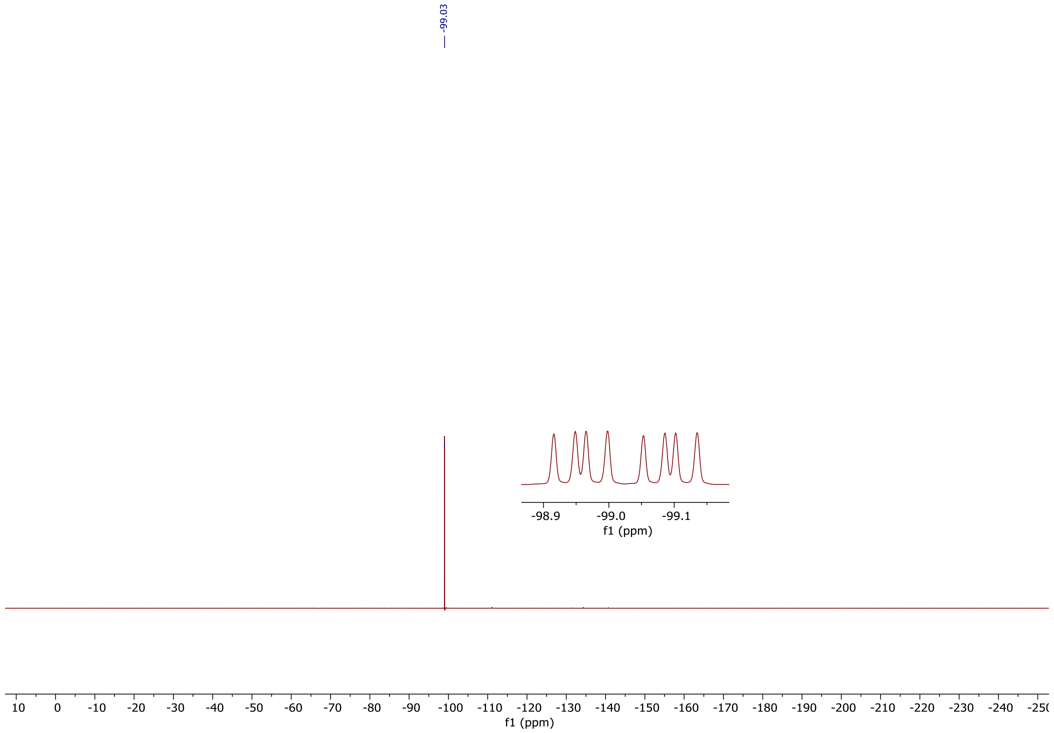

^{13}C NMR 14

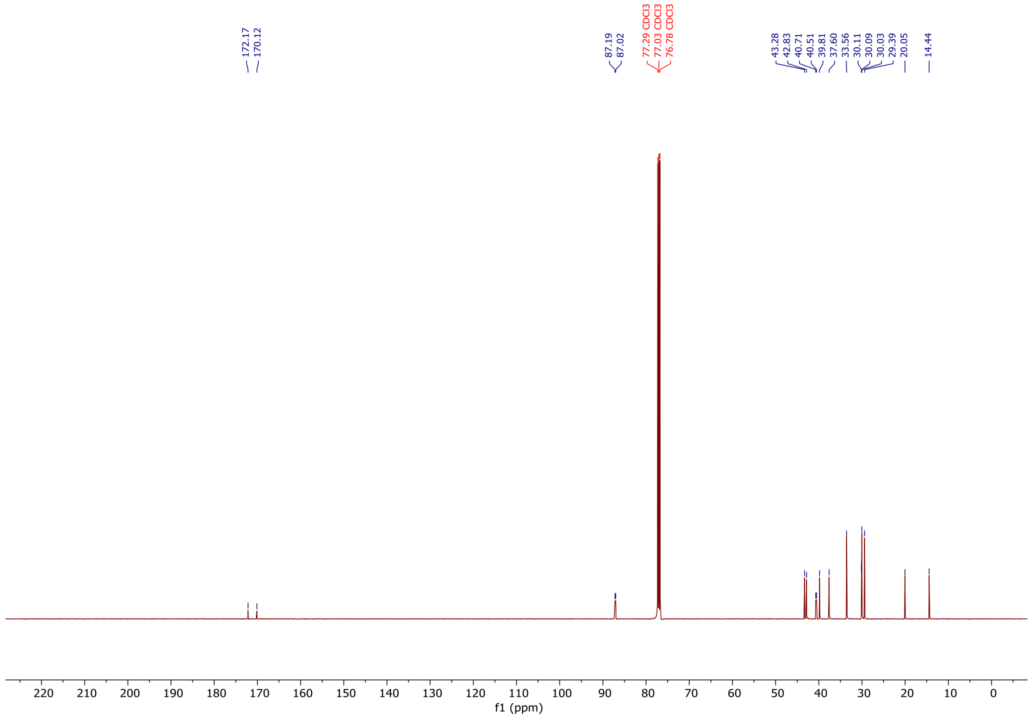

172.17
170.12
87.19
87.02
77.29 CDCl3
77.03 CDCl3
76.78 CDCl3
43.28
42.83
40.71
40.51
39.81
37.60
33.56
30.11
30.09
30.03
29.39
20.05
14.44
220 210 200 190 180 170 160 150 140 130 120 110 100 90 80 70 60 50 40 30 20 10 0
f1 (ppm)

^{1}H NMR 9

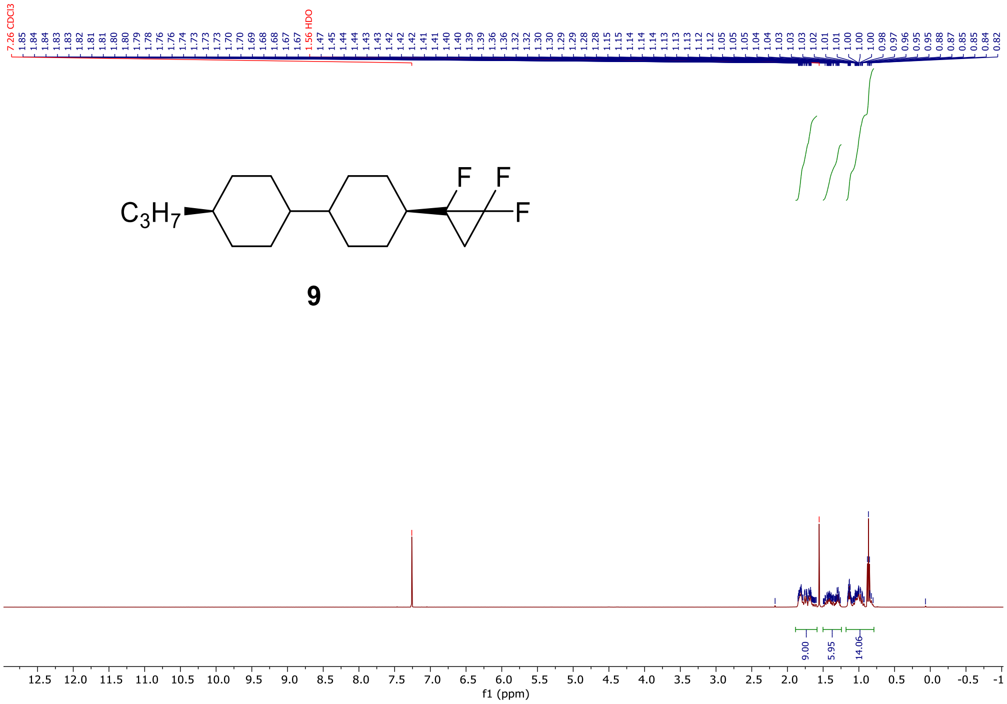

^{19}F {^{1}H} NMR 9

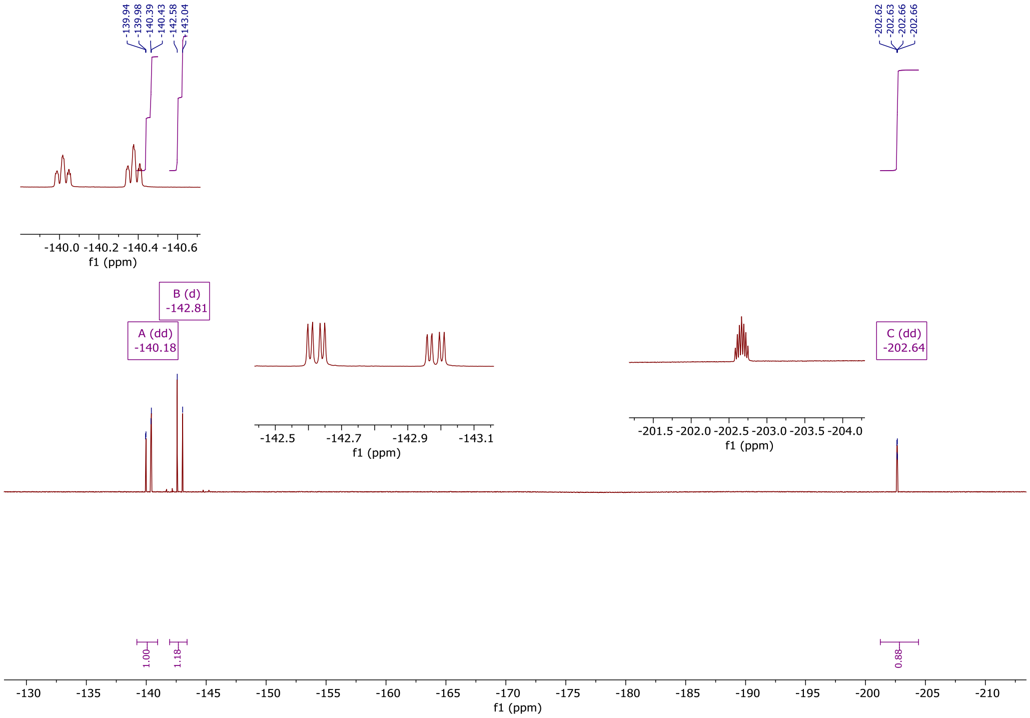

^{13}C NMR 9

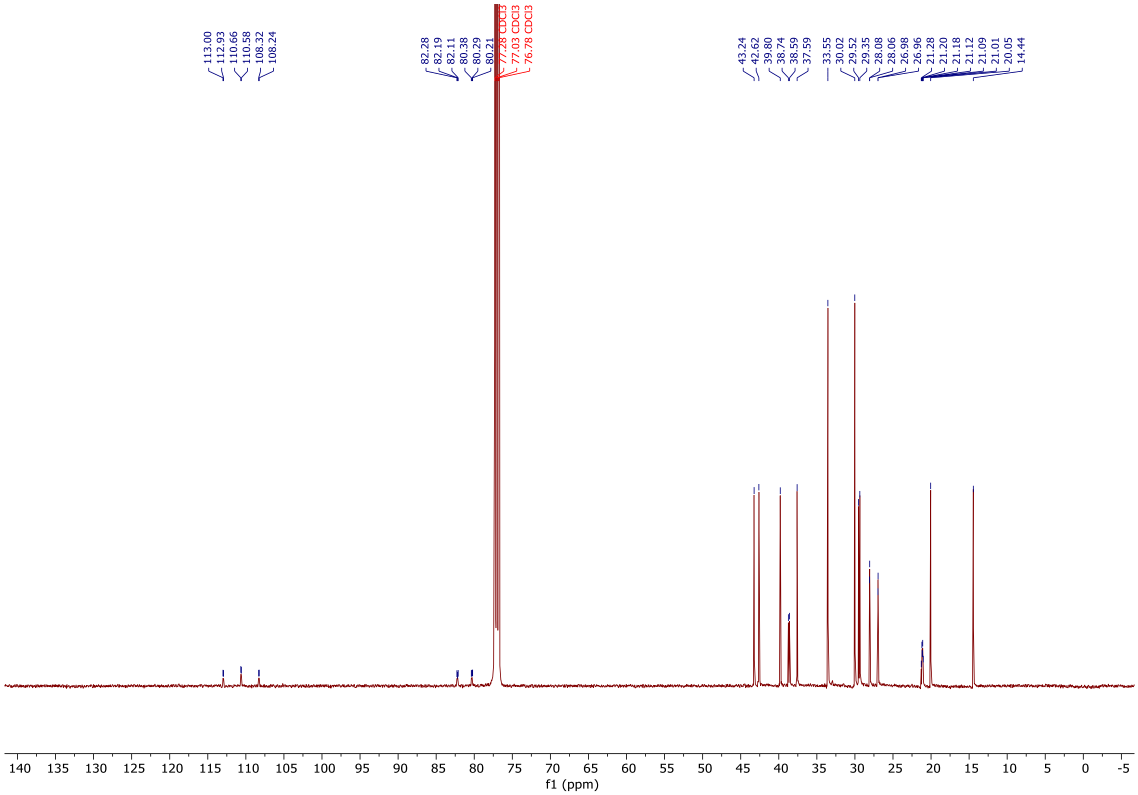

113.00
112.93
110.66
110.58
108.32
108.24
82.28
82.19
82.11
80.38
80.29
80.21
77.28 CDCl3
77.03 CDCl3
76.78 CDCl3
43.24
42.62
39.80
38.74
38.59
37.59
33.55
30.02
29.52
29.35
28.08
28.06
26.98
26.96
21.28
21.20
21.18
21.12
21.09
21.01
20.05
14.44
140 135 130 125 120 115 110 105 100 95 90 85 80 75 70 65 60 55 50 45 40 35 30 25 20 15 10 5 0 -5
f1 (ppm)

^{1}H NMR 17

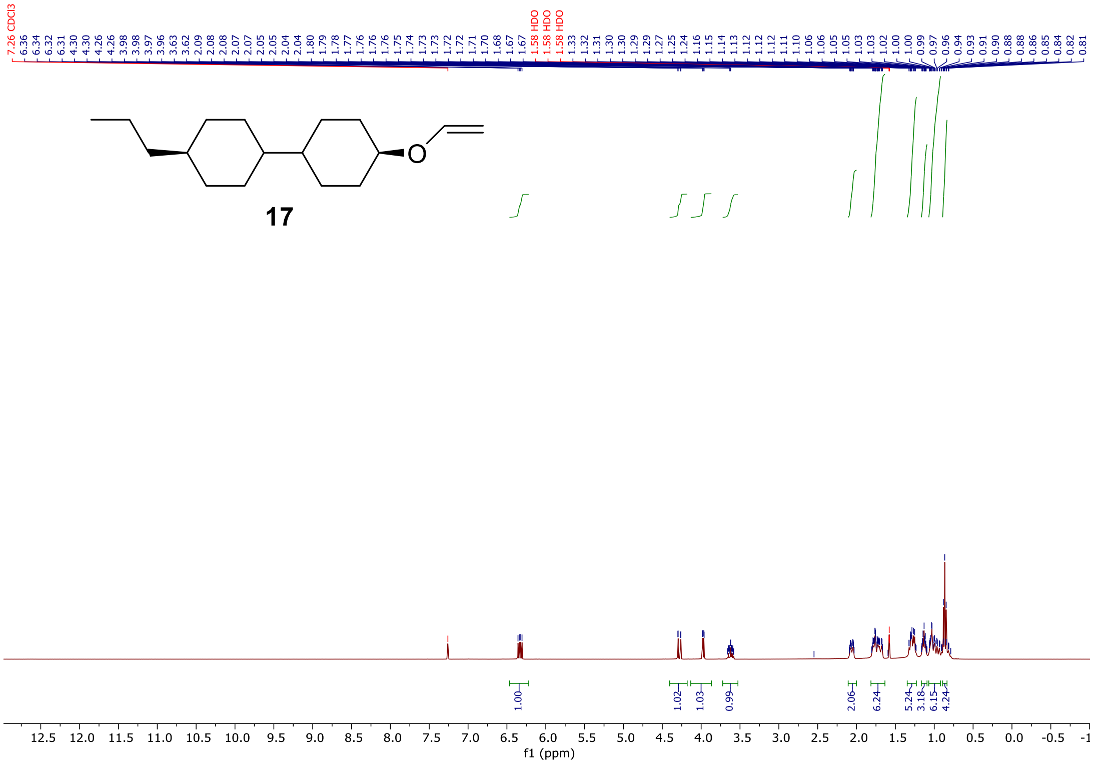

^{13}C NMR 17

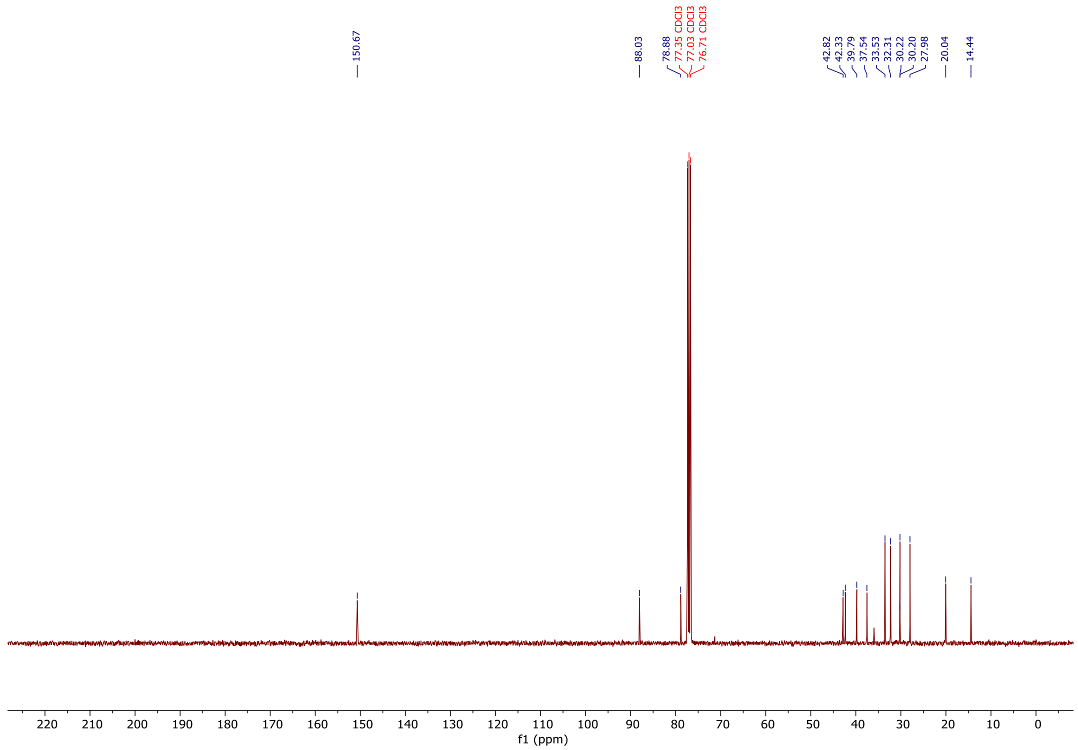

^{1}H NMR 10

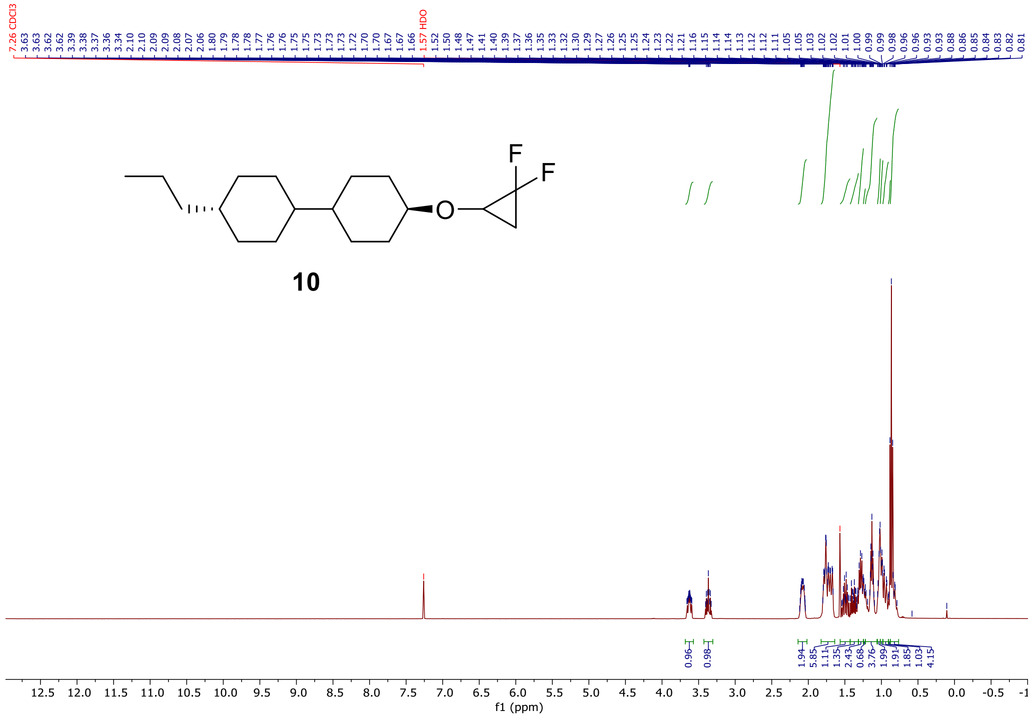

^{19}F {^{1}H} NMR 10

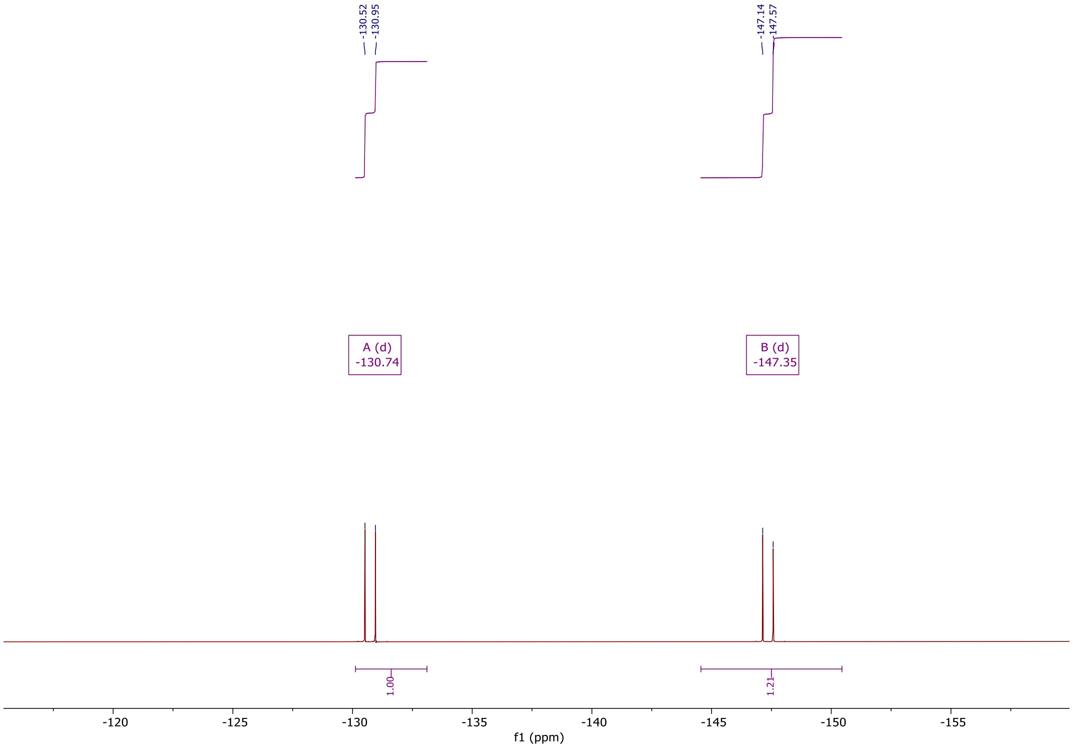

^{13}C NMR 10

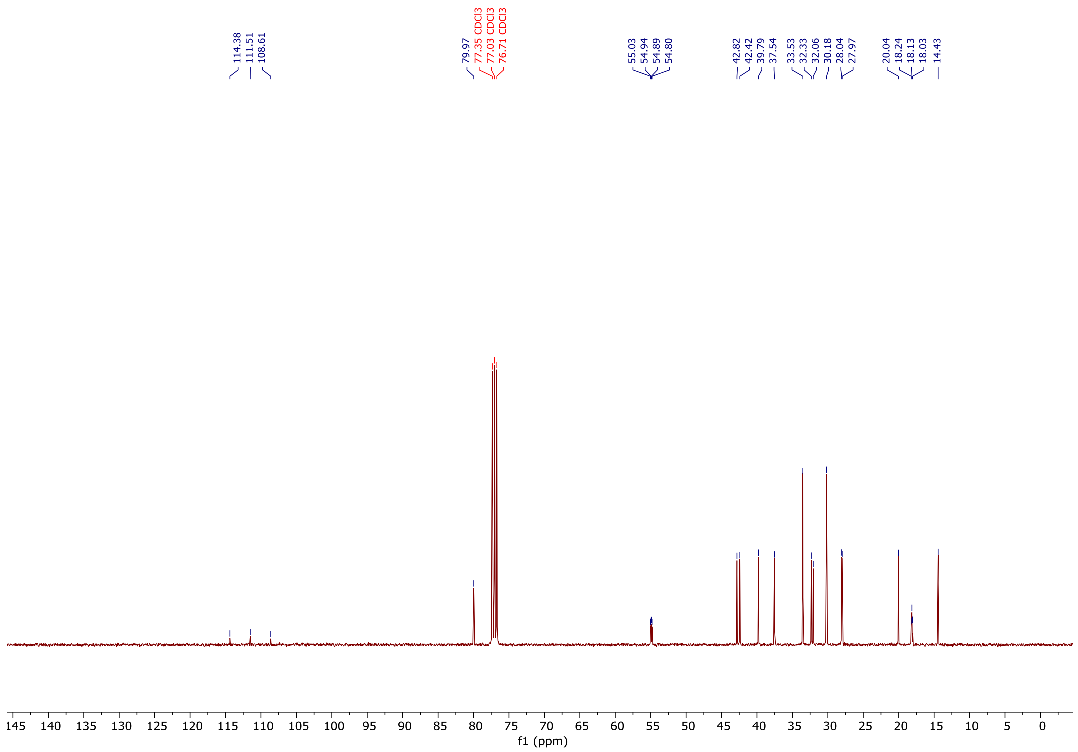
114.38
111.51
108.61
79.97
77.35 CDCl3
77.03 CDCl3
76.71 CDCl3
55.03
54.94
54.89
54.80
42.82
42.42
39.79
37.54
33.53
32.33
32.06
30.18
28.04
27.97
20.04
18.24
18.13
18.03
14.43
145 140 135 130 125 120 115 110 105 100 95 90 85 80 75 70 65 60 55 50 45 40 35 30 25 20 15 10 5 0
f1 (ppm)

^{1}H NMR 18

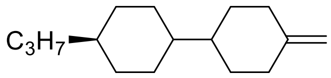

18

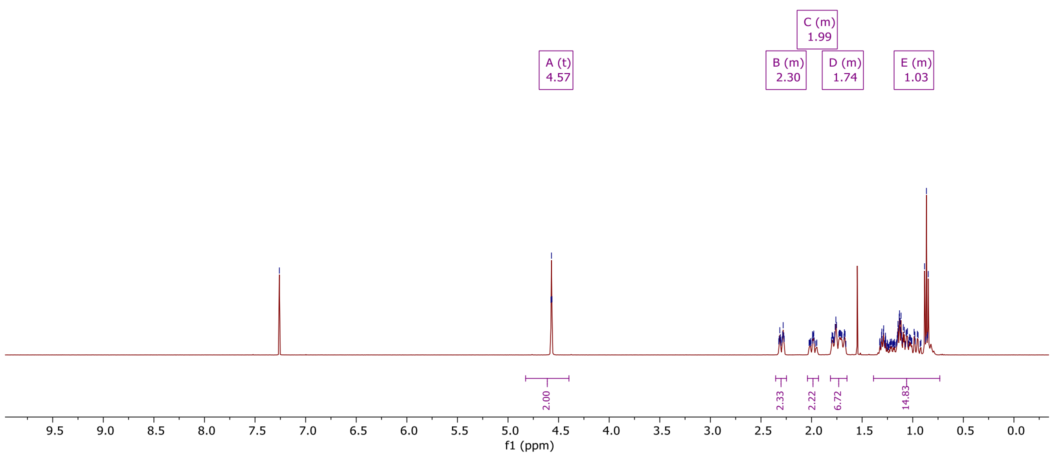

^{13}C NMR 18

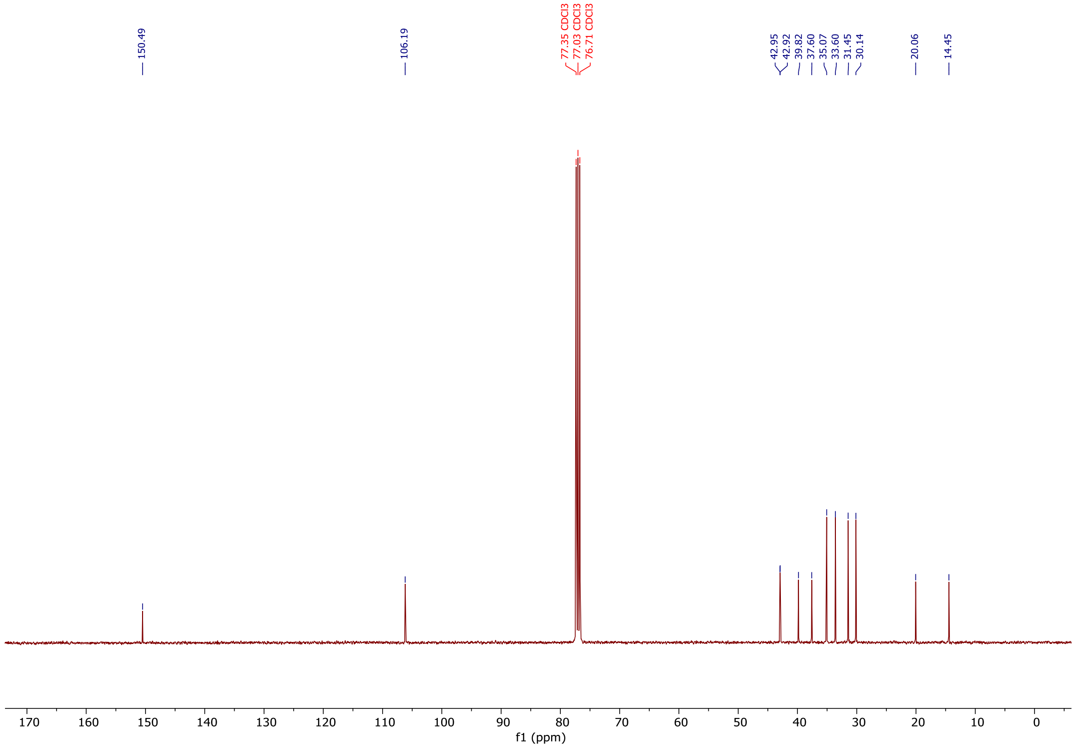

^{1}H NMR 11a

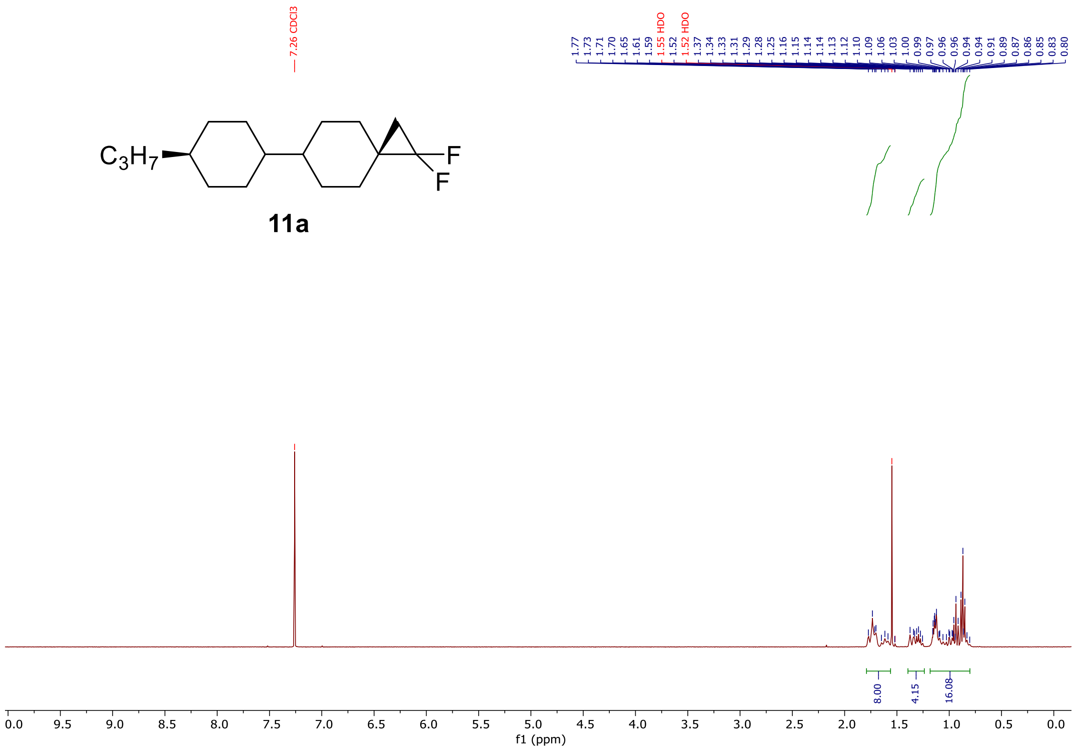

^{19}F {^{1}H} NMR 11a

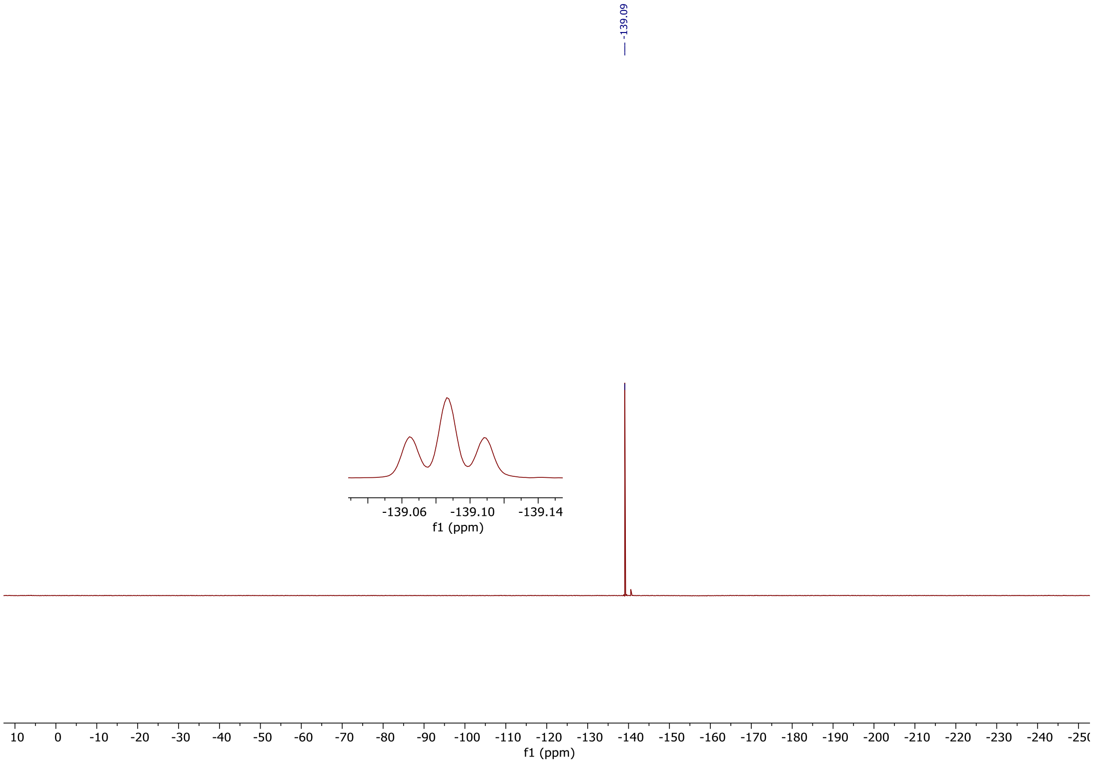

^{13}C NMR 11a

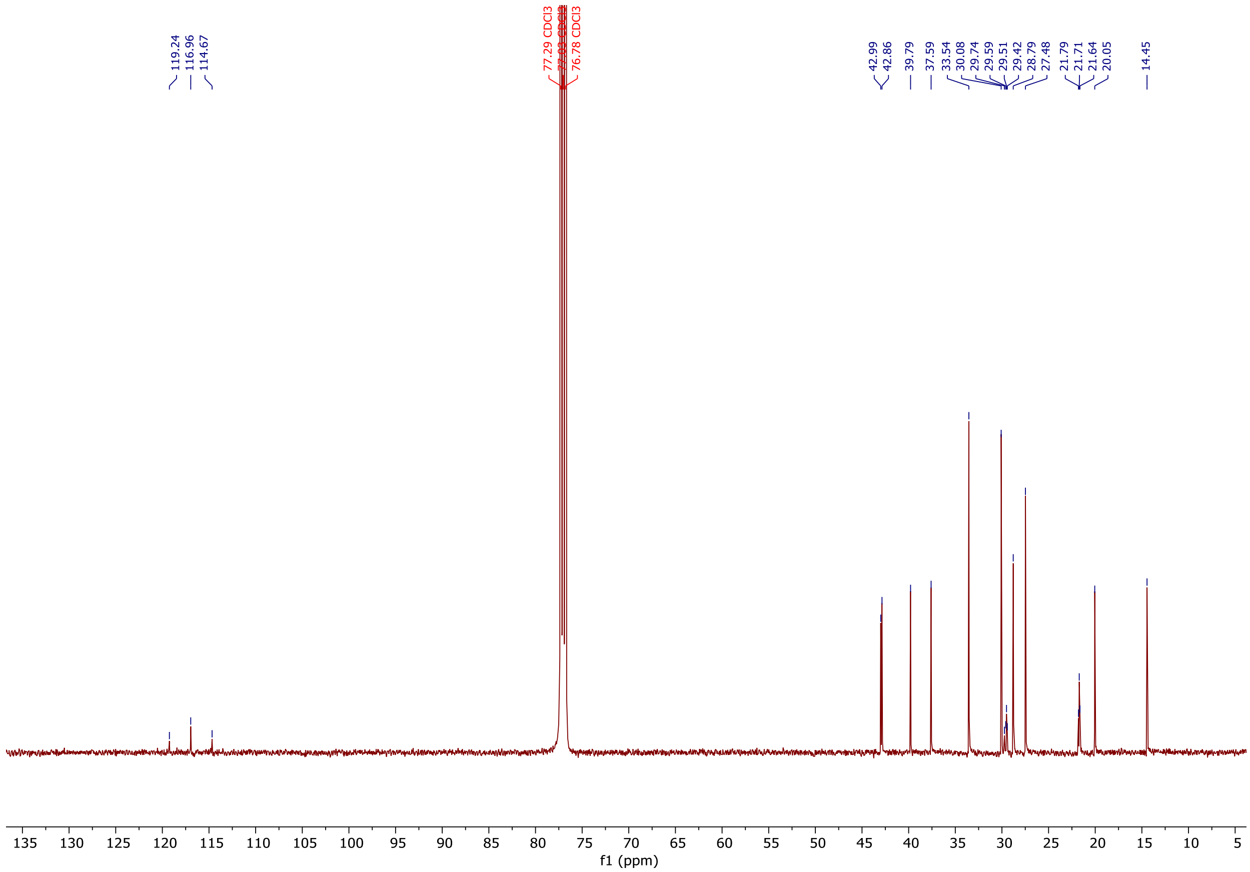

119.24
116.96
114.67
77.29 CDCl3
77.03 CDCl3
76.78 CDCl3
42.99
42.86
39.79
37.59
33.54
30.08
29.74
29.59
29.51
29.42
28.79
27.48
21.79
21.71
21.64
20.05
14.45
135 130 125 120 115 110 105 100 95 90 85 80 75 70 65 60 55 50 45 40 35 30 25 20 15 10 5
f1 (ppm)

Computational study

Gaussian 09[3] with the ωB97X-D density functional.[4]

The relative energies reported are the sum of the electronic energies at 0 K obtained with the 6-311+G(2d,p) basis set and the zero-point vibrational energies obtained with the 6-31G(d) basis set. (So they are not ΔG and not ΔH).

3 Gaussian 09, Rev. D.01, M. J. Frisch, G. W. Trucks, H. B. Schlegel, G. E. Scuseria, M. A. Robb, J. R. Cheeseman, G. Scalmani, V. Barone, B. Mennucci, G. A. Petersson, H. Nakatsuji, M. Caricato, X. Li, H. P. Hratchian, A. F. Izmaylov, J. Bloino, G. Zheng, J. L. Sonnenberg, M. Hada, M. Ehara, K. Toyota, R. Fukuda, J. Hasegawa, M. Ishida, T. Nakajima, Y. Honda, O. Kitao, H. Nakai, T. Vreven, J. A. Montgomery, J. E. Peralta, F. Ogliaro, M. Bearpark, J. J. Heyd, E. Brothers, K. N. Kudin, V. N. Staroverov, T. Keith, R. Kobayashi, J. Normand, K. Raghavachari, A. Rendell, J. C. Burant, S. S. Iyengar, J. Tomasi, M. Cossi, N. Rega, J. M. Millam, M. Klene, J. E. Knox, J. B. Cross, V. Bakken, C. Adamo, J. Jaramillo, R. Goperts, R. E. Stratmann, O. Yazyev, A. J. Austin, R. Cammi, C. Pomelli, J. W. Ochterski, R. L. Martin, K. Morokuma, V. G. Zakrzewski, G. A. Voth, P. Savador, J. J. Dannenberg, S. Dapprich, A. D. Daniels, O. Farkas, J. B. Foresman, J. V. Ortiz, J. Cioslowski, D. J. Fox, Gaussian Inc., Wallingford, CT, **2013**.

4 J.-D. Chai, M. Head-Gordon, *Phys. Chem. Chem. Phys.* **2008**, *10*, 6615-6620.

Relative Energies (kcal/mol; ωB97X-D/6-311+G(2d,p)//ωB97X-D/6-31G(d) + ZPE) **11a.**

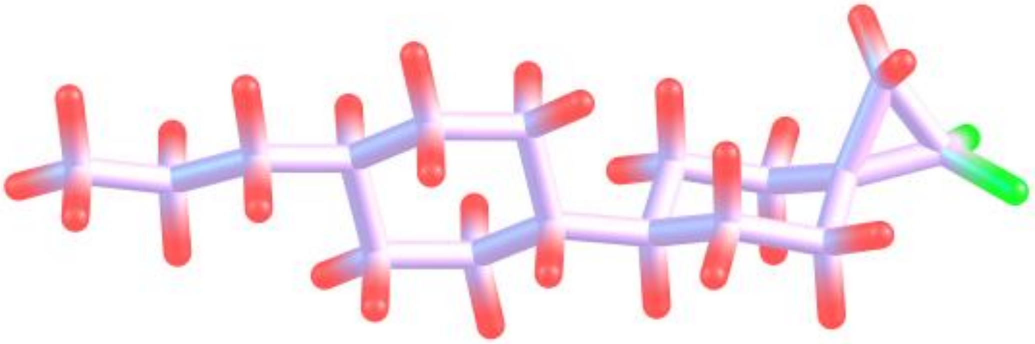

#1 0.00

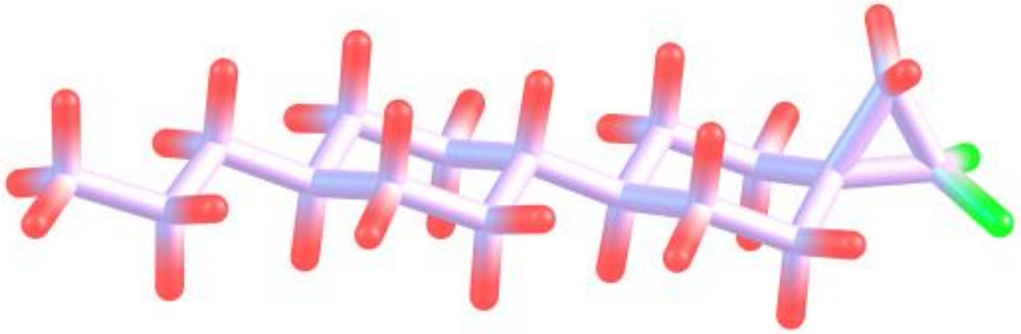

#2 0.10

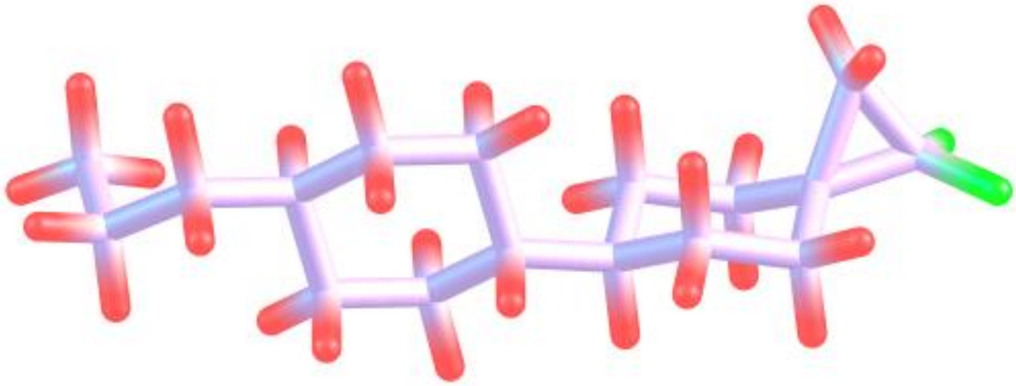

#3 0.43

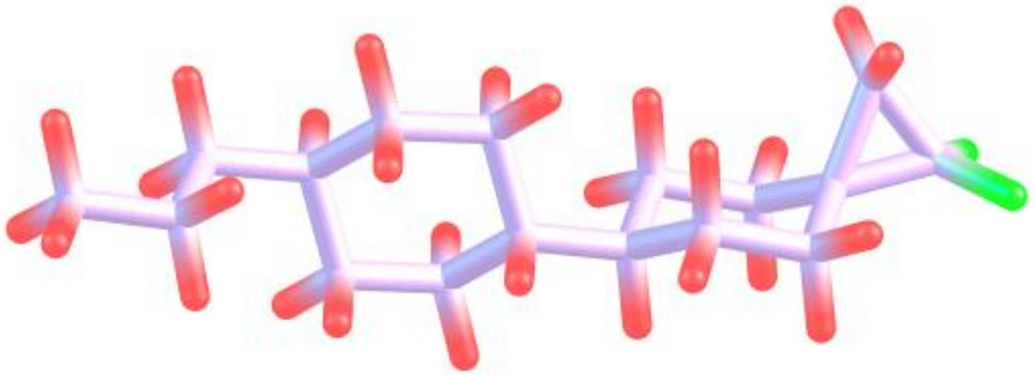

#4 1.02

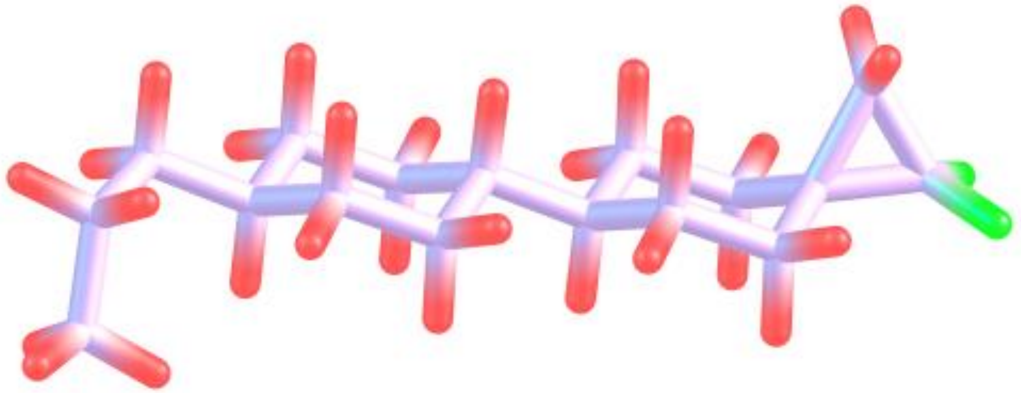

#5 0.52

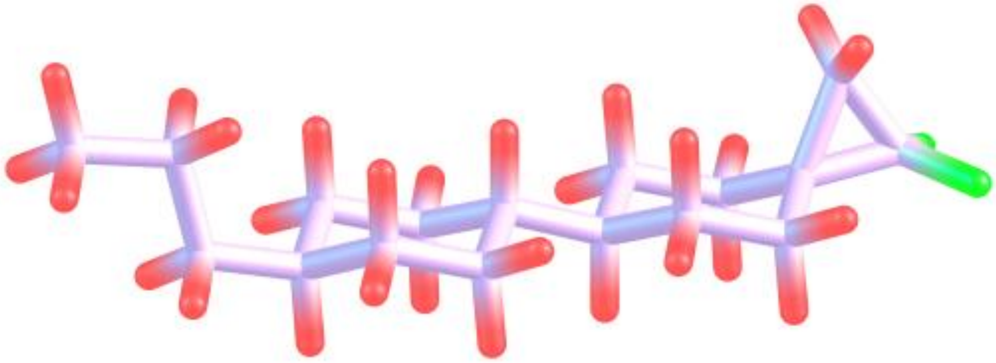

#6 1.12

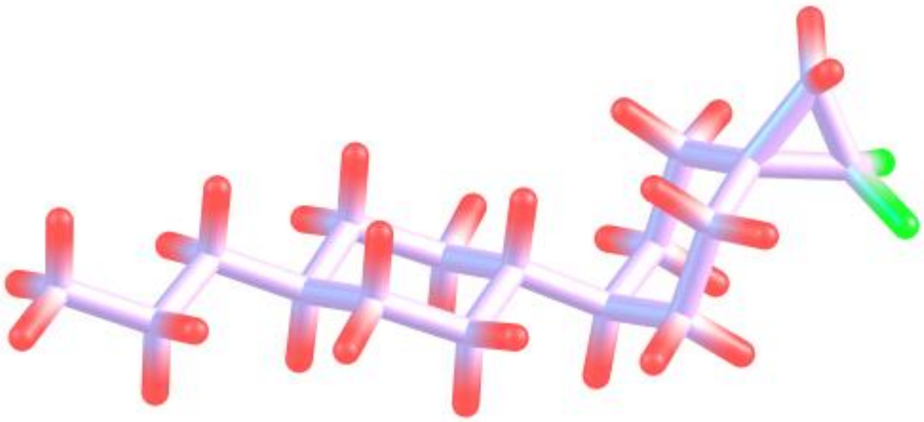

#7 1.25

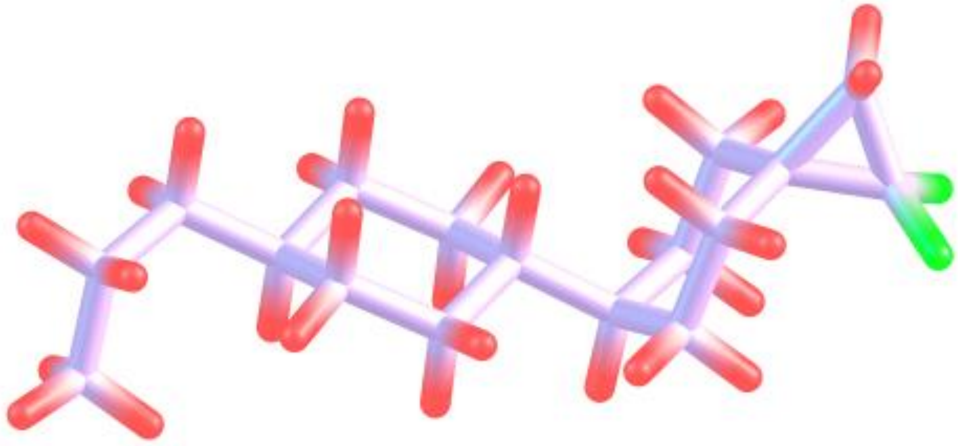

#8 1.19

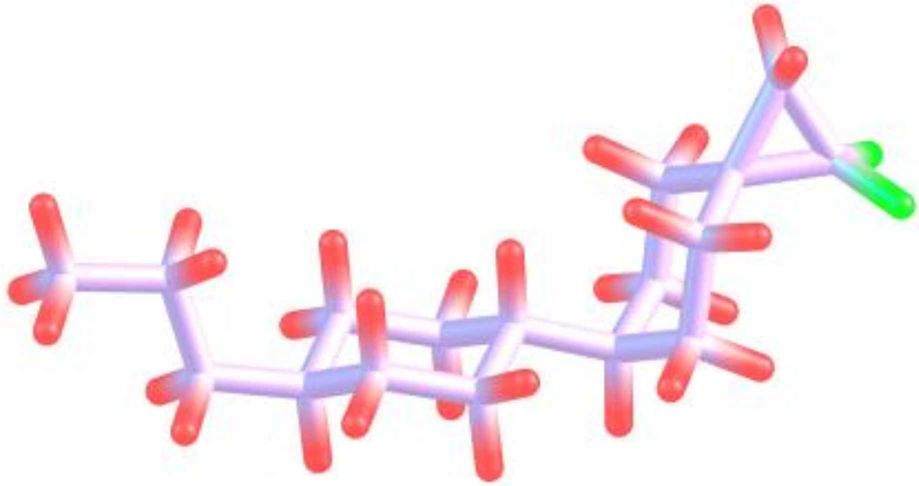

#9 1.86

Coordinates of #2 (Angstrom) for comparison with X-Ray crystal structure

1	-1.160789000	-1.963424000	0.182957000
6	-1.607064000	-1.051761000	-0.226596000
6	-3.765378000	0.298976000	-0.243526000
6	-1.493447000	1.451868000	-0.305503000
6	-2.944615000	1.522610000	0.173832000
6	-0.769751000	0.177959000	0.161130000
6	-3.059709000	-0.967420000	0.249414000
1	-3.785077000	0.265388000	-1.345274000
1	-1.472165000	1.484987000	-1.405557000
1	-2.959423000	1.599478000	1.271609000
1	-0.710599000	0.208566000	1.262230000
1	-3.084178000	-0.978273000	1.349990000
1	-1.590323000	-1.158439000	-1.322206000
1	-0.965816000	2.344691000	0.044557000
1	-3.414708000	2.437570000	-0.208269000
1	-3.595958000	-1.863278000	-0.083779000
6	0.676445000	0.100501000	-0.380551000
6	2.969685000	1.239204000	-0.434180000
6	2.829957000	-1.277091000	-0.472202000
6	3.613308000	-0.065235000	-0.012438000
6	1.383588000	-1.204741000	0.024013000
6	1.522805000	1.308119000	0.061963000
1	2.987077000	1.302658000	-1.530899000
1	2.840613000	-1.306492000	-1.570510000
1	1.372472000	-1.291031000	1.120820000
1	1.521685000	1.356721000	1.161025000
1	0.617692000	0.118963000	-1.482094000
1	3.540710000	2.095253000	-0.060843000
1	3.304871000	-2.201391000	-0.128551000
1	0.841471000	-2.070489000	-0.367208000
1	1.077067000	2.240685000	-0.296337000
6	5.081076000	-0.152773000	0.137294000
6	4.281241000	-0.124984000	1.384491000
1	4.192723000	-1.049863000	1.947222000
1	4.299558000	0.786566000	1.974913000
6	-5.211676000	0.411778000	0.250407000
1	-5.625154000	1.368601000	-0.099861000
1	-5.214311000	0.463830000	1.349670000
6	-6.138934000	-0.717895000	-0.202939000
1	-6.054723000	-0.839738000	-1.291476000
1	-5.814241000	-1.667605000	0.239122000
6	-7.597782000	-0.463500000	0.172595000
1	-8.243520000	-1.288334000	-0.146209000
1	-7.970600000	0.454612000	-0.295975000
1	-7.710075000	-0.350008000	1.257106000
9	5.851978000	0.909091000	-0.180059000
9	5.721047000	-1.288412000	-0.214319000

All Conformers, energies and dipole moments (kcal/mol and Hartree) **11a**

**Conf**	**Rel E**	SP	E1+ZPE	ZPE	Final E	Mux	Muy	Muz	Mu
**1**	**0.00**	-864.37288	-863.69818	0.43095	-863.94193	2.7327	-0.4293	-0.2777	2.7802
**2**	**0.10**	-864.37210	-863.69799	0.43033	-863.94177	-2.7015	0.0913	0.6618	2.7829
**3**	**0.43**	-864.37283	-863.69754	0.43160	-863.94124	-2.7929	-0.5326	0.3612	2.8661
**4**	**1.02**	-864.37184	-863.69662	0.43154	-863.94030	-2.7282	-0.2693	0.3426	2.7628
**5**	**0.52**	-864.37224	-863.69736	0.43113	-863.94110	-2.7449	0.252	0.7908	2.8677
**6**	**1.12**	-864.37107	-863.69641	0.43094	-863.94014	0.4746	2.7256	0	2.7667
**7**	**1.25**	-864.37156	-863.69639	0.43162	-863.93994	-2.1869	-0.0344	1.0486	2.4255
**8**	**1.19**	-864.37132	-863.69658	0.43130	-863.94003	-2.1446	0.2862	1.2746	2.5111
**9**	**1.86**	-864.37020	-863.69551	0.43123	-863.93897	0.8974	2.2397	0	2.4128
**10**	**2.09**	-864.36968	-863.69490	0.43109	-863.93859	2.7884	-0.6269	-0.3696	2.8818
**11**	**3.29**	-864.36867	-863.69293	0.43198	-863.93669	-2.5431	0.2152	1.1126	2.7841
**12**	**2.33**	-864.36926	-863.69449	0.43104	-863.93822	-2.7525	0.3298	0.7941	2.8837
**13**	**2.71**	-864.36904	-863.69400	0.43143	-863.93761	2.8455	0.0758	0.5773	2.9045
**15**	**3.59**	-864.36829	-863.69253	0.43208	-863.93621	-2.5357	0.5587	1.2305	2.8733
**16**	**3.01**	-864.36859	-863.69360	0.43146	-863.93713	-2.2338	-0.0673	1.038	2.4641
**17**	**3.30**	-864.36835	-863.69321	0.43167	-863.93668	2.1953	0.1647	1.2376	2.5255
**19**	**3.92**	-864.36748	-863.69230	0.43180	-863.93569	2.378	0.8808	-0.1851	2.5426

All Conformers, energies and dipole moments (kcal/mol and Hartree) **11b**

Conf	Rel E	SP	E1+ZPE	ZPE	Final E	Mux	Muy	Muz	Mu
**2**	**0.00**	-864.37271	-863.69854	0.43056	-863.94215	-0.6681	0.2166	-2.2999	2.4048
**1**	**0.04**	-864.37357	-863.69857	0.43148	-863.94209	-0.4327	-2.2596	0.3942	2.3342
**3**	**0.20**	-864.37338	-863.69840	0.43155	-863.94183	0.5197	-2.2494	-0.3252	2.3315
**4**	**0.60**	-864.37206	-863.69781	0.43086	-863.94120	0.3568	2.1091	0.8943	2.3185
**5**	**0.64**	-864.37303	-863.69753	0.43190	-863.94113	-0.8751	-0.2953	-2.1549	2.3445
**6**	**0.72**	-864.37144	-863.69752	0.43043	-863.94101	-0.4279	-0.0001	-2.2892	2.3289
**7**	**1.14**	-864.37099	-863.69654	0.43066	-863.94034	-1.5171	-2.1596	0.9247	2.7965
**8**	**1.94**	-864.37108	-863.69528	0.43202	-863.93906	-1.7525	1.4726	1.5339	2.7555
**9**	**2.09**	-864.36973	-863.69512	0.43091	-863.93882	-2.4159	1.2403	0	2.7157
**10**	**2.10**	-864.37012	-863.69538	0.43131	-863.93881	0.5355	-2.0788	-0.7064	2.2599
**12**	**2.44**	-864.37000	-863.69468	0.43173	-863.93827	-0.9	-0.1844	-2.2077	2.3912
**13**	**2.59**	-864.36932	-863.69471	0.43128	-863.93803	0.522	2.0116	0.8046	2.2286
**11**	**3.17**	-864.36893	-863.69359	0.43183	-863.93710	1.1766	0.3554	2.0861	2.4212
**15**	**3.29**	-864.36864	-863.69343	0.43172	-863.93692	1.4077	-0.1542	1.9224	2.3876

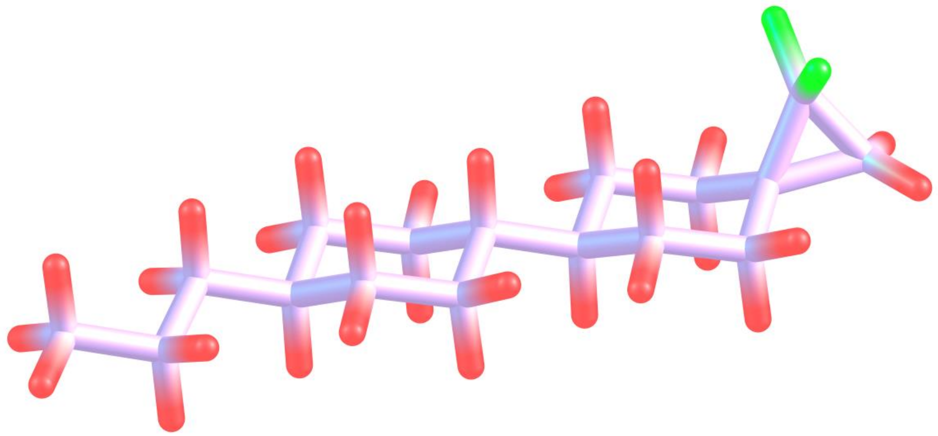

#2 0.00

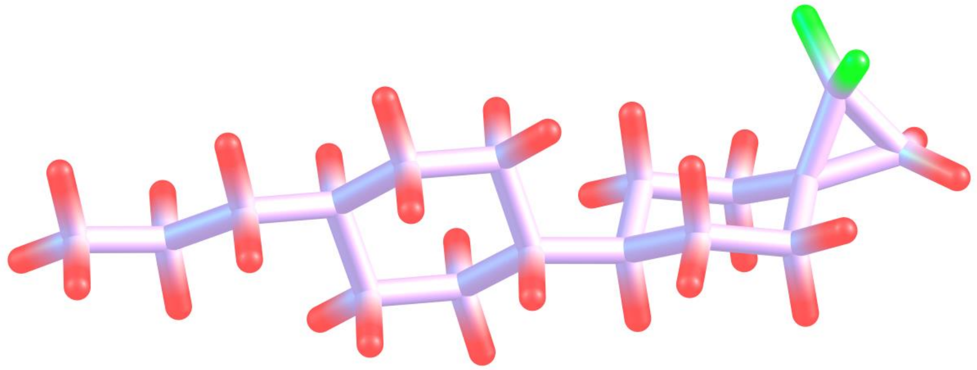

#1 0.04

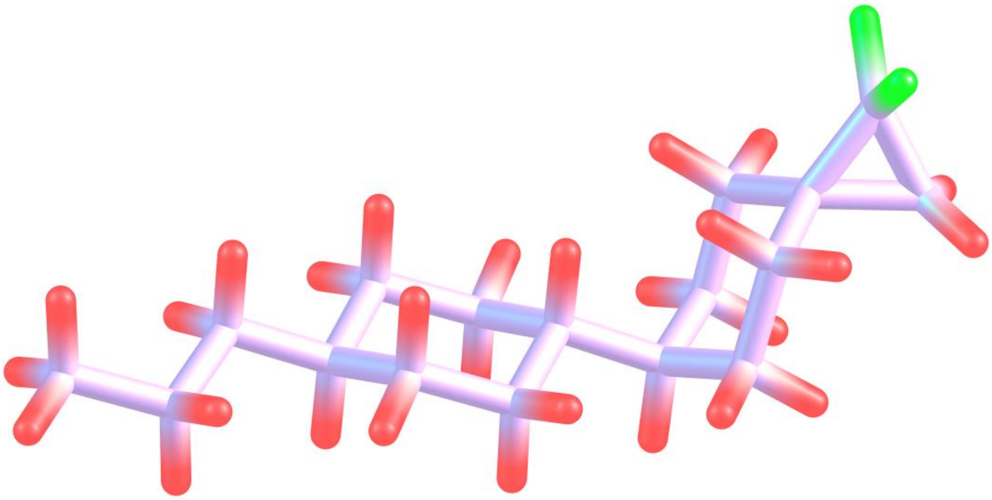

#7 1.14

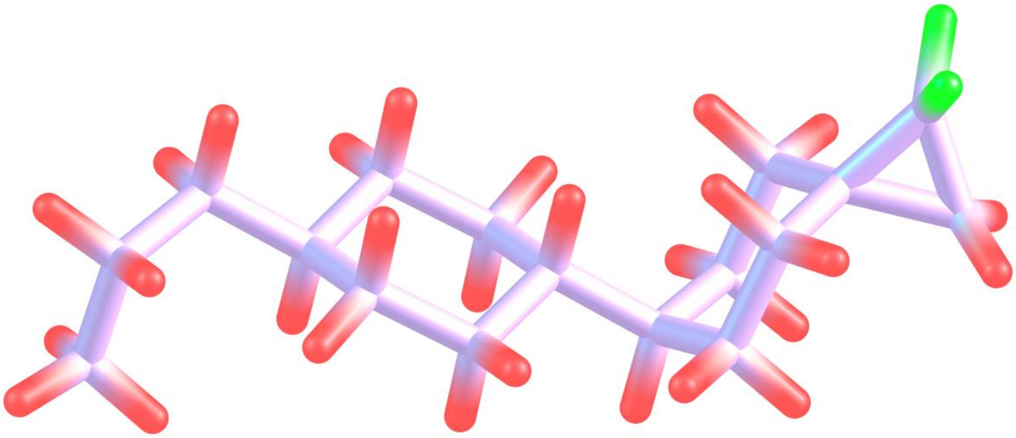

#8 1.94

Single crystal X-ray structure analysis

X-ray diffraction data for **11a** was collected at 125 K using a Rigaku MM-007HF High Brilliance RA generator/confocal optics with XtaLAB P200 diffractometer [Cu Kα radiation (λ = 1.54187 Å)]. Intensity data were collected using ω steps accumulating area detector. Data were collected using CrystalClear[5] and processed (including correction for Lorentz, polarization and absorption) using CrysAlisPro.[6]
Structures were solved by dual-space (SHELXT[7]) methods and refined by full-matrix least-squares against F2 (SHELXL-2018/3[7].) Selected crystallographic data are presented below in Table 1. The data can be obtained free of charge from The Cambridge

Crystallographic Data Centre via www.ccdc.cam.ac.uk/structures. (CCDC 1981334)

*5. CrystalClear-SM Exper*t v2.1. Rigaku Americas, The Woodlands, Texas, USA, and Rigaku Corporation, Tokyo, Japan, 2015.

6. CrysAlisPro v1.171.39.8d. Rigaku Oxford Diffraction, Rigaku Corporation, Oxford, U.K. 2015.

7. Sheldrick, G. M. *Acta Crystallogr., Sect. C.* **2015**, *71*, 3-8.

Table 1 (Crystal data)

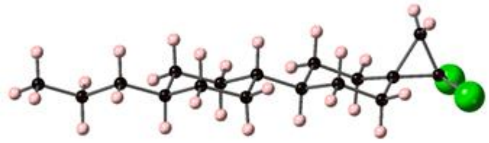

Chemical Formula: $C_{17}H_{28}F_2$

Display Formula: $C_{17}H_{28}F_2$

Spacegroup: $P\bar{1}$

Crystal system: Triclinic

a: 5.9533(14)Å

b: 10.8810(2) Å

c: 13.1778(3)Å

α: 113.800(2)°

β: 90.4969(19)°

γ: 98.612(18)°

V: 769.98(3) $Å^3$

Z value : 2

Crystal Colour, Habit: colourless, prism

F_{000} : 296.00

***ρ* (calc) [g/cm³]:** 1.166

Reflections collected: 8735

Images for DSC analysis and POM

Compound 8

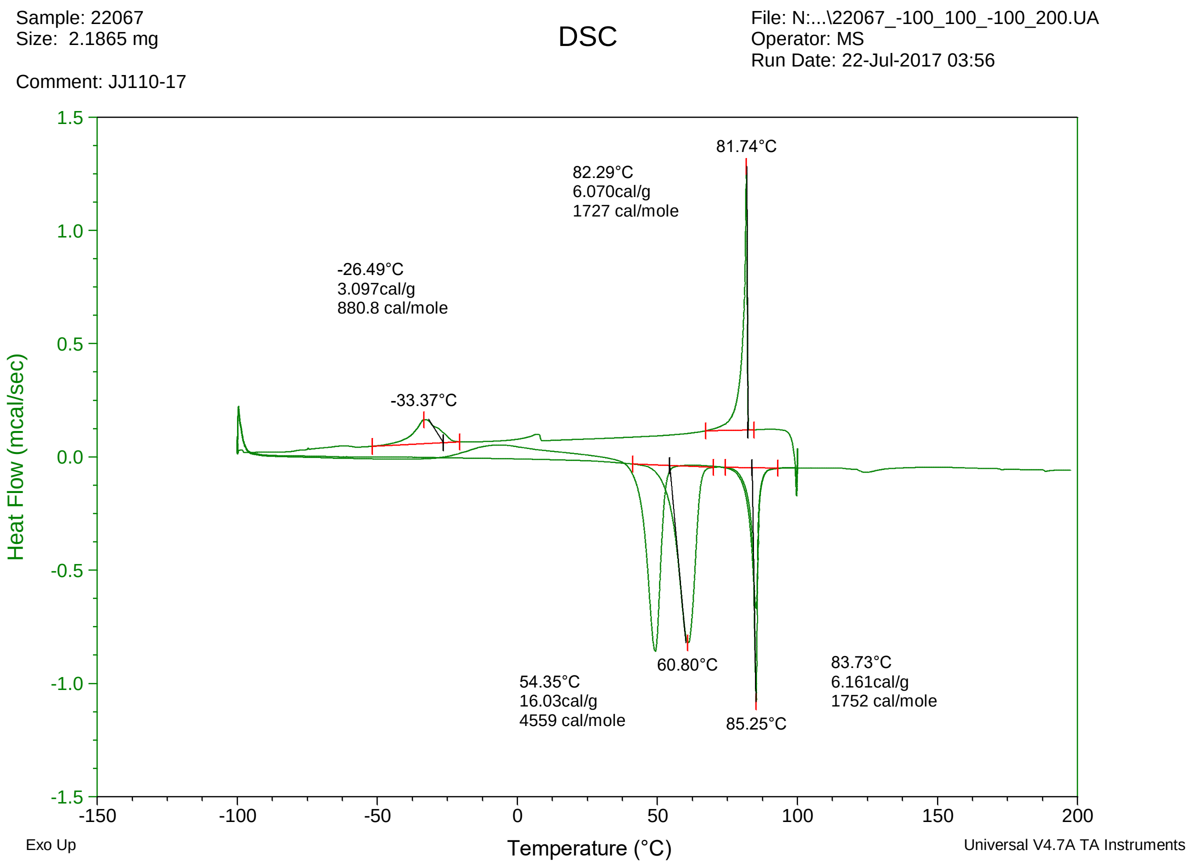

POM

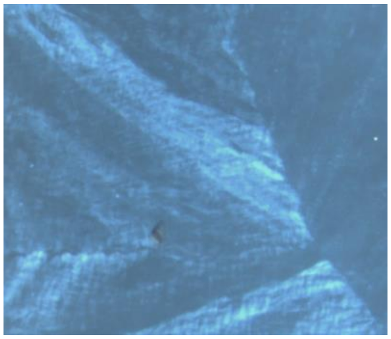

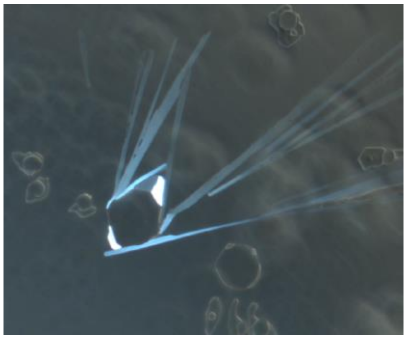

Compound 9

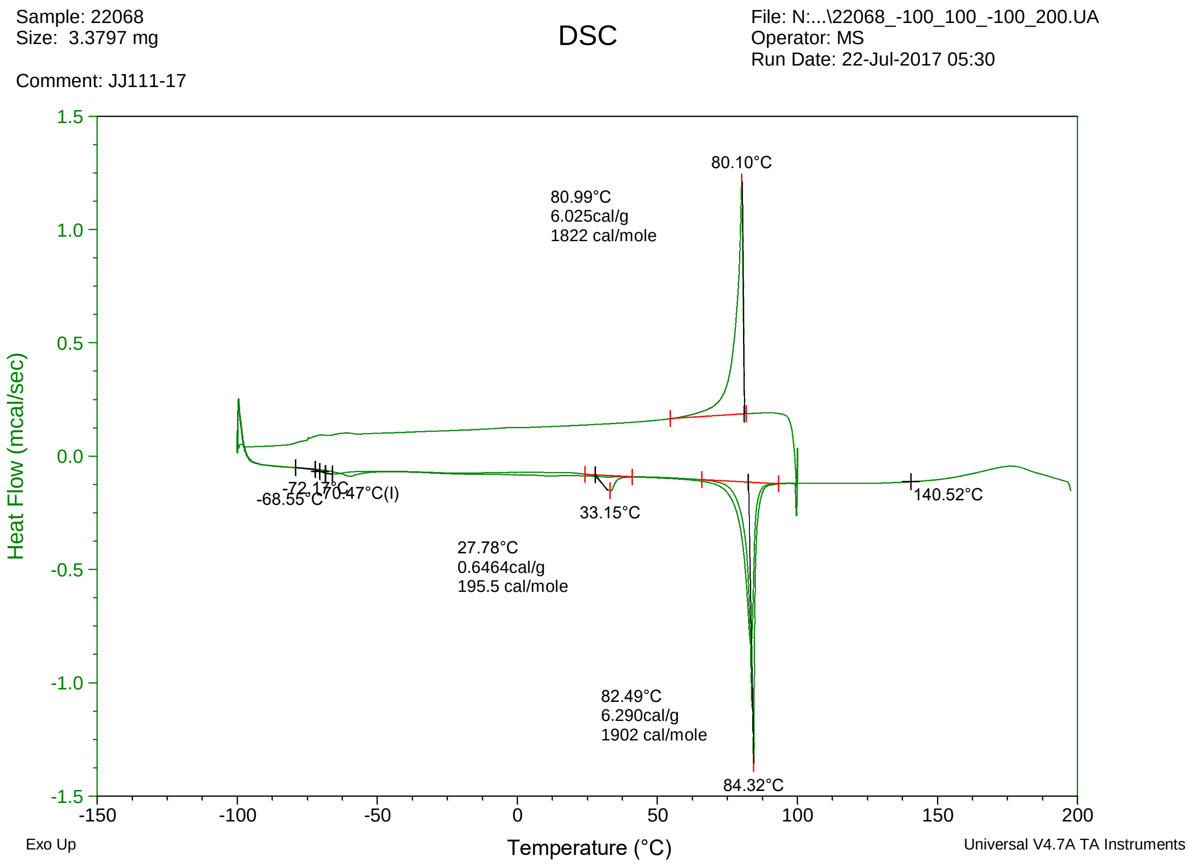

POM

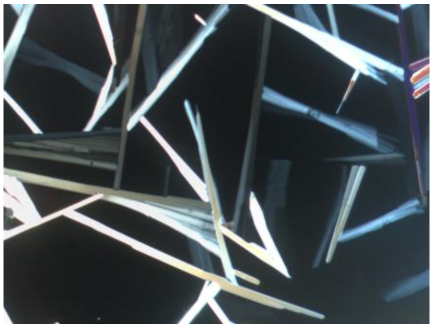

Compound 10

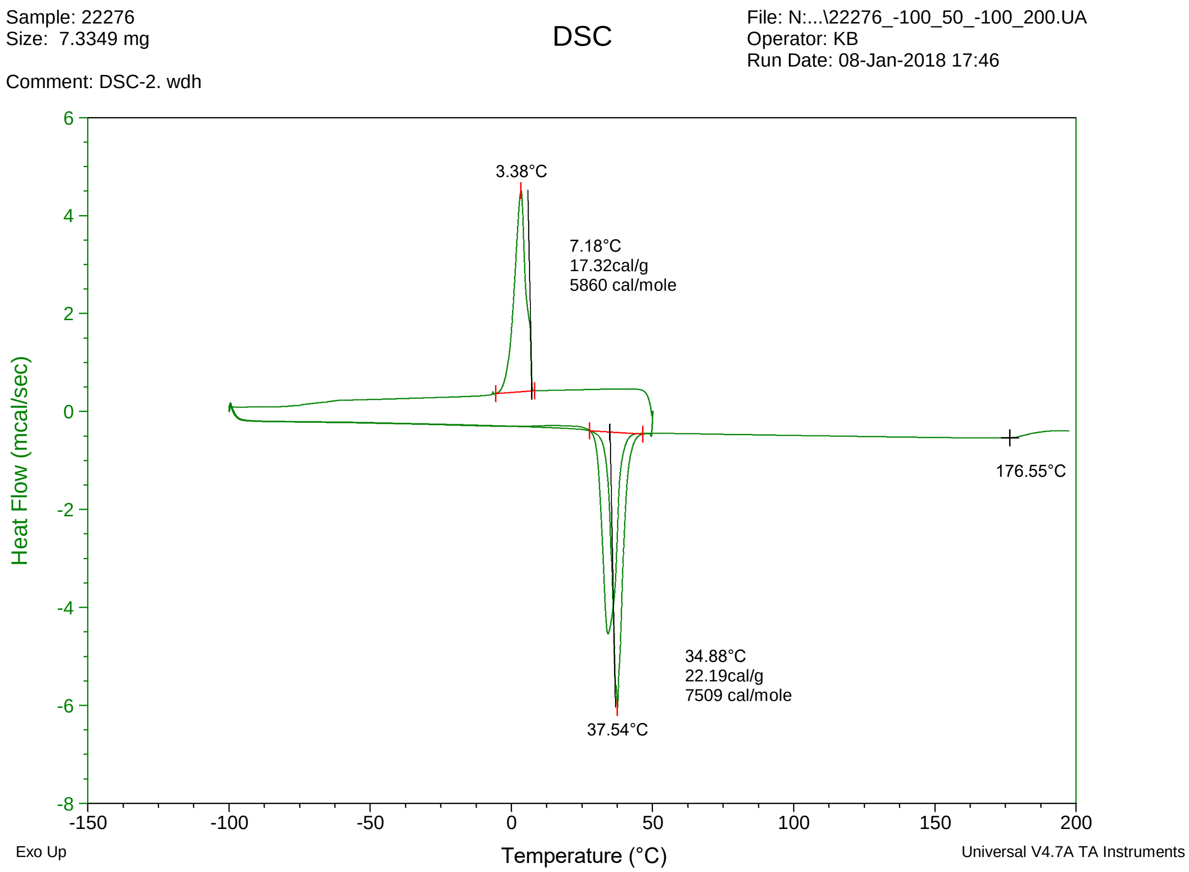

POM

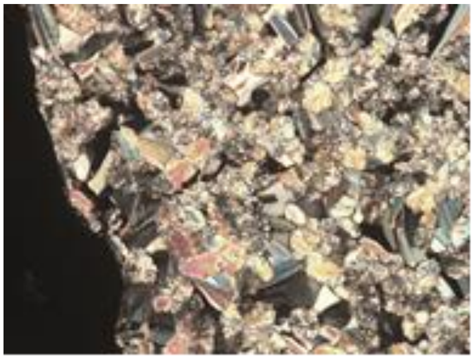

Compound 11

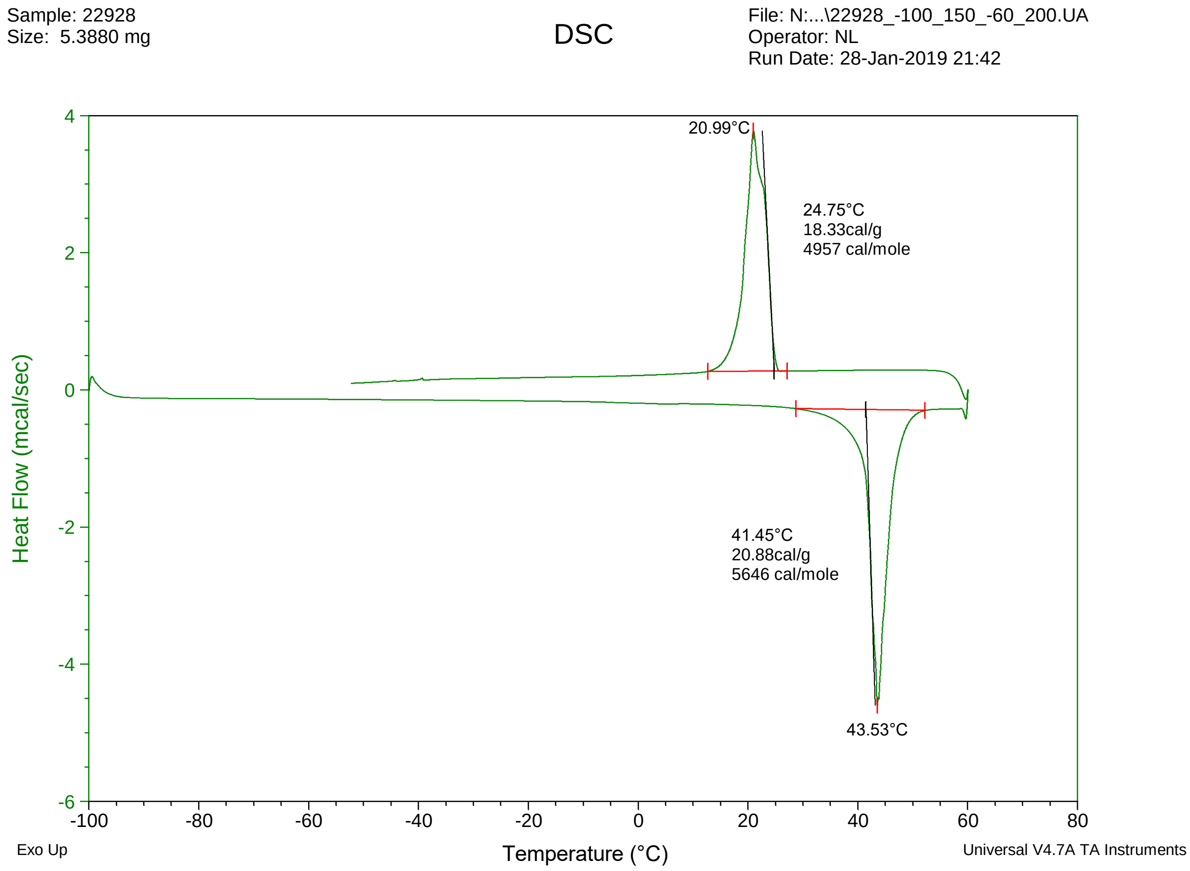

POM

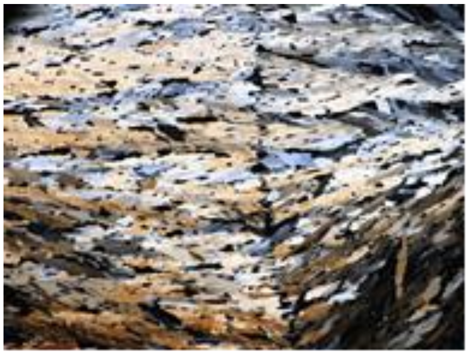

Supplement: File 1 — Experimental protocols and NMR spectra. [file Beilstein_J_Org_Chem-16-674-s001.pdf]
